# Genomic introgressions from African rice (*Oryza glaberrima*) in Asian rice (*O. sativa*) lead to the identification of key QTLs for panicle architecture

**DOI:** 10.1186/s12864-023-09695-6

**Published:** 2023-10-04

**Authors:** Hélène Adam, Andrés Gutiérrez, Marie Couderc, François Sabot, Fabrice Ntakirutimana, Julien Serret, Julie Orjuela, James Tregear, Stefan Jouannic, Mathias Lorieux

**Affiliations:** 1 UMR DIADE, University of Montpellier, IRD, Cirad, Montpellier, France; 2 Agrobiodiversity Unit, Alliance Bioversity-CIAT, Cali, Colombia

**Keywords:** *Oryza glaberrima*, *Oryza sativa*, Chromosome segment substitution lines, Panicle architecture, QTL, Genomic variation

## Abstract

**Background:**

Developing high yielding varieties is a major challenge for breeders tackling the challenges of climate change in agriculture. The panicle (inflorescence) architecture of rice is one of the key components of yield potential and displays high inter- and intra-specific variability. The genus Oryza features two different crop species: Asian rice (*Oryza sativa* L.) and the African rice (*O.* *glaberrima* Steud.). One of the main morphological differences between the two independently domesticated species is the structure (or complexity) of the panicle, with *O. sativa* displaying a highly branched panicle, which in turn produces a larger number of grains than that of *O.* *glaberrima*. The gene regulatory network that governs intra- and interspecific panicle diversity is still under-studied.

**Results:**

To identify genetic factors linked to panicle architecture diversity in the two species, we used a set of 60 Chromosome Segment Substitution Lines (CSSLs) issued from third generation backcross (BC_3_DH) and carrying genomic segments from *O.* *glaberrima* cv. MG12 in the genetic background of *O. sativa* Tropical Japonica cv. Caiapó. Phenotypic data were collected for rachis and primary branch length, primary, secondary and tertiary branch number and spikelet number. A total of 15 QTLs were localized on chromosomes 1, 2, 3, 7, 11 and 12, QTLs associated with enhanced secondary and tertiary branch numbers were detected in two CSSLs. Furthermore, BC_4_F_3:5_ lines carrying different combinations of substituted segments were produced to decipher the effects of the identified QTL regions on variations in panicle architecture. A detailed analysis of phenotypes versus genotypes was carried out between the two parental genomes within these regions in order to understand how *O.* *glaberrima* introgression events may lead to alterations in panicle traits.

**Conclusion:**

Our analysis led to the detection of genomic variations between *O. sativa* cv. Caiapó and *O.* *glaberrima* cv. MG12 in regions associated with enhanced panicle traits in specific CSSLs. These regions contain a number of key genes that regulate panicle development in *O. sativa* and their interspecific genomic variations may explain the phenotypic effects observed.

**Supplementary Information:**

The online version contains supplementary material available at 10.1186/s12864-023-09695-6.

## Background

Improving or enhancing the sustainability of rice yield continues to be a crucial challenge in the breeding of this crop, especially in the context of a growing world population and with regard to climate change. Inflorescence architecture in rice directly affects yield potential through the regulation of grain number. Grain number per panicle depends on orders and numbers of branches (i.e., primary, secondary and potentially tertiary branches as well as both lateral and terminal spikelets) and on the length of each axis. Rice panicle architecture is based on the production of a series of lateral meristems with distinct identities [1]. After the floral transition, the Shoot Apical Meristem (SAM) is converted into an indeterminate Rachis Meristem (RM) which produces several axillary meristems, the primary branch meristems (PBMs) that subsequently give rise to primary branches (PBs). Once the RM has lost its activity, the newly formed PBM elongates and initiates a variable number of axillary meristems. These acquire the identity of secondary branch meristems (SBMs) and develop into secondary branches (SBs), which in turn may produce tertiary branch meristems (TBMs), or alternatively differentiate directly into lateral spikelet meristem (SpMs) and then florets. The basic architecture of the panicle is thus determined by patterns of axillary meristem formation and the specification of their identities. Overall, the complexity and diversity of panicle branching can be considered as governed by two key elements: the number of axillary meristems produced during the indeterminate phase; and the rate of meristem fate transition, which determines whether an axillary meristem grows into a higher-order branch or differentiates into a spikelet.

Over recent decades, a number of genes and quantitative trait loci (QTLs) associated with panicle development and affecting its architecture have been identified and functionally described in rice [2–8]. Regulatory genes influencing grains number per panicle include *Gn1a/OsCKX2* (GA20-oxidase1, LOC_Os03g63970 [9]), *IPA1/WFP* (*OsSPL14* SQUAMOSA PROMOTER BINDING PROTEIN-LIKE, LOC_Os08g39890 [10, 11]), *LAX1* (basic-helix-loop-helix, LOC_Os01g61480 [12]), *PAP2* (MADS box, LOC_Os03g54170 [13, 14]), *DEP1* (G protein gamma subunit, LOC_Os09g26999, [15]), MOC1 (GRAS family nuclear protein, LOC_Os06g40780 [16]), *TAW1* (ALOG, LOC_Os10g33780 [17]), *qSrn7/FZP* (AP2, LOC_Os07g47330 [18–21]) and *APO1* (F-box protein, LOC_Os06g45460 [22]), which positively regulate the numbers of primary and secondary branches. Many of these genes and QTLs encode transcriptions factors, such as those belonging to the SQUAMOSA PROMOTER BINDING PROTEIN-LIKE (SPL), APETALA2/ETYLENE RESPONSE FACTOR (AP2/ERF), MADS-BOX, HOMEOBOX and ALOG families, and are implicated in axillary meristem formation or in the conversion of indeterminate meristems to spikelets, thus influencing the branching complexity of the panicle [12, 13, 20, 23, 24]. For example, the SQUAMOSA PROMOTER BINDING PROTEIN-LIKE *OsPL14* (also known as IDEAL PLANT ARCHITECTURE1, IPA1 and WHEALTHY FARMERS's PANICLE, WFP) promotes panicle branching [10, 11], while FRIZZY PANICLE (FZP also known as qSNR7), an ethylene-responsive element binding factors, promotes SM identity [20, 25]. Variations in their regulation lead to significant effects on panicle architecture [10, 19, 26].

Most studies of rice panicle development have focused on the Asian crop plant *Oryza sativa* L*.* However, a second species of cultivated rice, *O.* *glaberrima* Steud, was domesticated independently of Asian rice, in the inner delta of Niger river [27] from the wild relative species *O.* *barthii* around 2,500 years ago. Cultivation of *O. sativa* was introduced into West Africa around 400 years ago and this crop has since largely replaced *O.* *glaberrima* – although the latter is still grown, it represents only 1–2% of the total cultivated area. *O. glaberrima* is more adapted to a variety of ecologically and climatically diverse regions, its agronomically useful characters including higher tolerance to drought, high temperatures and infertile soils as well as a greater resistance to various biotic stresses [28–33]. While *O. sativa* is less adapted to the African environment, this species has a higher yield potential than *O. glaberrima*, the difference being partly explained by the higher panicle complexity of Asian rice [34]. Even if molecular pathways associated with the regulation of axillary meristem identity seem to be conserved between the two species, it was possible to identify a set of genes displaying differential expression in relation to panicle phenotypic variability between African and Asian rice [34]. Moreover, a Genome-Wide Association Study (GWAS) carried out on a panel of African rice accessions allowed the identification of several new genomic regions associated with panicle branching diversity and climatic variables [35].

In the context of climate change, achieving high tolerance to environmental stresses is of paramount importance to rice agriculture while knowledge relating to the genetic control of traits governing yield potential in African rice remains very limited. Agronomic traits such as heading date, yield, plant height and grain size are controlled by many genes exerting major and/or minor effects. Identifying the genes and genomic regions associated with interspecific variation in the number of grains produced per panicle is challenging due to the polygenic nature and environmental sensitivity of panicle branching traits.

Libraries of introgression lines (ILs) provide a useful means to investigate genetic phenomena in an interspecific context. An ILs library is a collection of lines with a common genetic background (i.e. the recipient genome), each carrying one or a few genomic regions originating from a donor genome. The ILs are generally obtained through recurrent backcrossing onto the recipient parent, followed by several rounds of self-fertilization (BC_n_F_n_) or double haploidization (BC_n_DH). The genome of the donor genotype is then represented in the library by discrete homozygous chromosomal fragments. Such ILs are also called Chromosome Segment Substitution Lines (CSSLs). In contrast to segregating populations, such as Recombinant Inbred Lines (RILs), Doubled Haploid (DH), Backcross (BC) or F_2:3_ populations, QTL analyses using CSSLs are suitable to evaluate minor allelic differences conferred by additive QTLs in a uniform genetic background in populations of small sizes [36, 37]. Genetic dissection of complex traits can thus be achieved by combining genetic variation with introgressed genomic fragments so as to reduce interference effects between QTLs. Hence, CSSLs provide a powerful means to identify QTLs for complex traits with minor and/or additive effects [38, 39]. This approach may provide access to novel and potentially beneficial genes "hidden" in the genetic background of a related species that can be discovered when placed in the genetic background of a cultivated species.

In this study, we evaluated several panicle morphological traits in a BC_3_DH CSSL population developed from an interspecific cross between the recipient parent *O. sativa ssp. japonica* (cv. Caiapó) and the donor parent *O. glaberrima* (cv. MG12) [40]. QTLs relating to modifications in panicle morphology were mapped on the rice genome, and 4^th^ generation backcross (BC_4_F_3:5_) generations were produced to evaluate the effect of each region on panicle phenotype variation. Finally, DNA polymorphisms between the two parental genomes were investigated in detail for some key genes that were previous implicated in panicle branching diversity in *O. sativa*. The results obtained will help allow the identification of functionally significant polymorphisms and, more generally, provide an insight into the genetic bases of panicle architecture diversity between the two species.

## Results

### Panicle traits in the CSSL population

In order to identify new genetic factors governing the panicle branching diversity observed between *O. sativa* cv. Caiapó (hereinafter referred to as *Os_Caiapó*) and *O. glaberrima* cv. MG12 (hereinafter referred to as *Og_MG12*), phenotypic measurements were performed on a population of 60 BC_3_DH CSSLs and their parents (Additional file 1: Table S2) [40].

**Fig. 1:**
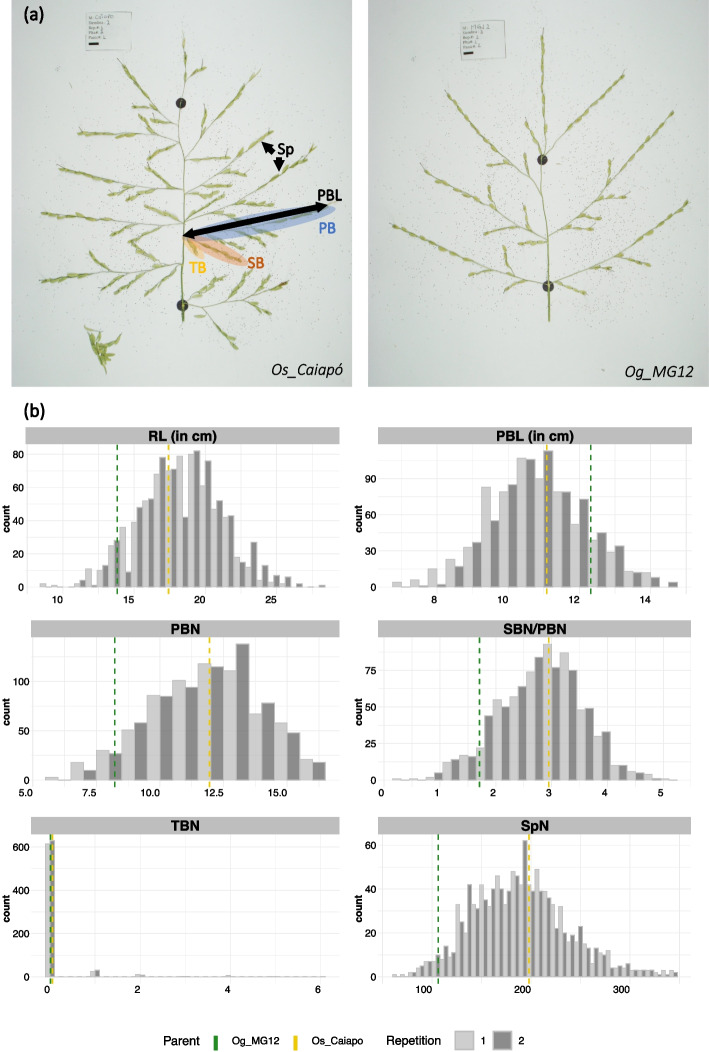
Panicle structure and morphological panicle trait values in the two parental lines and the 60 BC3DH CSSLs. **a** Contrasted spread panicle between the two parental lines (*Os_Caiapó* and *Og_MG12*)*.* Panicle traits measured are showing in the image: the rachis is the main and central axis of the panicle, rachis length (RL) is measured between the 2 dot black points. The primary branches (PB) (in blue) are axis attached to the rachis, the primary branches bear secondary branches (SB) (in red) which bear tertiary branches (TB) (in yellow). Spikelets (Sp) are attached to the branches. The Primary branch Length (PBL) is the average of the primary branch lengths in the panicle. **b** Distribution of panicle trait values in the 60 BC3DH CSSLs. Dashed vertical lines correspond to the mean values of each parent (green for *Og_MG12* and yellow for *Os_Caiapó*). Values for repetitions 1 and 2 are shown in light gray and dark gray, respectively. Abbreviations: RL, Rachis Length; PBN, Primary Branch Number; PBL, Primary Branch Length; SBN, Secondary Branch Number; TBN, Tertiary Branch Number; SpN, Spikelet Number

Six quantitative traits were evaluated per panicle: rachis length (RL); primary, secondary and tertiary branch numbers (PBN, SBN, TBN respectively); primary branch length average (PBL); and spikelet number (SpN) (Table 1, Additional file 1: Table S2). The two parents showed contrasting panicle phenotypes with a higher panicle complexity observed in *Os_Caiapó* compared to *Og_MG12*. More specifically, the *Os_Caiapó* parent displayed panicles with more PBN and SBN leading to a higher SpN compared to *Og_MG12* (Fig. 1a). In contrast, the *Og_MG12* parent produced panicles with longer PBs compared to *Os_Caiapó*. Since the trials were conducted as two repeated experiments, we computed broad sense heritability for each experiment (Table 1). All the measured variables showed broad sense heritability values higher than 0.8 except for TBN. For all traits, the mean of the CSSL population was similar to the mean of the recurrent *Os_Caiapó* parent (Table 1). The coefficient of variation for the SBN/PBN ratio was higher in the CSSL population than in *Os_Caiapó* (26.13% versus 18.26%). Histograms of the CSSLs for panicle traits showed a continuous distribution for all traits except TBN (Fig. 1b). The observed distributions were similar between the two repeated experiments. The abnormal distribution of the TBN trait was associated with a high value of CV (Coefficient Variation) and a low heritability value. This observation results from the rare and unstable nature of this trait which is dependent on environmental conditions and does not appear frequently in the *Os_Caiapó* parent. Moreover, the formation of tertiary branches has not been described previously in *O*. *glaberrima* populations [35], nor was it observed in our earlier experiments.

**Table 1:** Variation, descriptive statistics and broad sense heritability of the scored traits in the CSSL population and the parents

	**Parentals**										**60 CSSLs**					
	***Os*_Caiapó (Recurrent Parent)**					***Og*_MG12**										
**Traits**	**Mean**	**Std**	**CV (%)**	**Min**	**Max**	**Mean**	**Std**	**CV (%)**	**Min**	**Max**	**Mean**	**Std**	**CV (%)**	**Min**	**Max**	**H2**
**RL**	17.41	2.19	12.55	13.0	23.0	13.81	2.46	17.84	8.8	19.2	18.52	2.72	14.71	8.7	28.2	0.86
**PBN**	11.94	2.11	17.66	8.0	16.0	8.22	1.44	17.46	5.0	13.0	12.05	1.99	16.48	6.0	16.0	0.89
**PBL**	11.08	1.0	9.017	8.3	12.93	12.32	1.65	13.36	8.71	15.3	10.78	1.31	12.06	6.99	14.66	0.86
**SBN/PBN**	2.95	0.514	18.25	1.5	3.78	1.71	0.57	33.40	0.0	3.43	2.83	0.74	26.13	0.36	5.13	0.89
**TBN**	0.06	0.23	418.1	0.0	1.0	0.01	0.12	848.53	0.55	1.0	0.12	0.54	438.05	0.0	6.0	0.53
**SpN**	197.94	44.78	22.62	98.0	291.0	106.24	26.08	24.54	62.0	164.0	193.51	45.89	23.72	83.0	348.0	0.86

*Abbreviations*: *RL* rachis length in cm, *PBN* primary branch number per panicle, *PBL* primary branch length average in cm/panicle, *SBN/PBN* secondary branch number per primary branch per panicle, *TBN* tertiary branch number per panicle, *SpN* spikeletnumber per panicle, *Std* standart deviation, *CV* coeficient variation, *Min* minimum, *Max* maximum, *H*2 heritability value

**Fig. 2:**
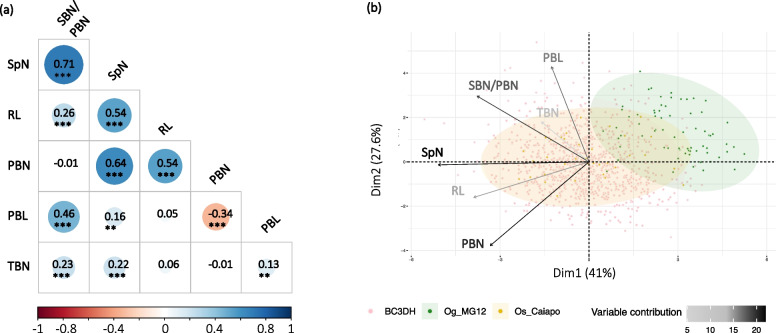
Description of the morphological panicle traits observed in the BC3DH CSSL lines and parents. **a** Correlation plot of panicle morphological traits based on the Pearson method between panicle traits, ** represents the *p*-values of Pearson correlation coefficients < 0.01; *** < 0.001. **b** Principal component analysis with trait contributions and individual panicle distributions. Abbreviations: RL, Rachis Length; PBN, Primary Branch Number; PBL, Primary Branch Length; SBN, Secondary Branch Number; TBN, Tertiary Branch Number; SpN, Spikelet Number

By analyzing relationships between the different panicle traits, we observed the highest correlation (0.71) between SpN and SBN/PBN (Fig. 2a). Other traits were moderately correlated with each other, notably PBN and SpN. We also observed a low correlation between PBL and SBN. Principal component analysis showed a separate distribution of *Os_Caiapó* and *Og_MG12* parents on the PC1 and PC2 axes. In contrast, we observed an overlapping of the BC_3_DH lines with the *Os_Caiapó* recurrent parent (Fig. 2b). Analysis of variable contributions showed that SpN is the main trait contributing to the diversity observed in this population.

### Detection of QTLs associated with panicle architecture traits

A Dunnett's test revealed a total of 37 lines with significant differences compared to the *Os_Caiapó* recurrent parent for all traits combined (Additional file 1: Table S4). In general, these lines bear at least two *Og_MG12* introgression fragments. For this reason, assigning the underlying QTLs to a single chromosome segment was not straightforward. The CSSL Finder software was therefore used to detect genomic regions associated with panicle phenotype variation with an F-test at each marker [40]. Graphical genotype representations were also produced in which the CSSLs were ordered by trait value for each evaluated trait (Additional file 2: Figure S1). We considered the F-test as significant when its value was higher than 10.0 ($p-value<\sim 0.002 based on Bonferron{i}{\prime}s test correlation 0.05/200$). QTLs were assigned to the *Og_MG12* introgressed regions of these CSSLs if the F-test was significant and confirmed by the graphical genotype logical analysis. In total, 15 QTLs were detected for all combined traits analyzed with the exception of rachis length (Table 2, Additional file 2: Fig. S1, S2). The F-test value of the markers allowing the detection of each QTL are reported in Table 2 along with their positions in the *O. sativa* and *O. glaberrima* reference genomes (cv. Nipponbare IRGSP-1.0 and cv. CG14 OglaRS2 respectively).

**Table 2:** QTLs for morphological panicle traits detected in the CSSL population

Traits	ID_QTL	Chrom	SSR Marker	F-Test value	Significance F-Test	R^2^ value	Additivity	p(F-test)	-LOG10(p)	Effects compared to the recurrent parent *Os_Caiapó*	Position (bp) in *O. sativa* Caiapó	Position (bp) in *O. glaberrima* MG12	Position (bp) in *O. sativa* MSU 7	Position (bp) in *O. glaberrima* OMAPv2	Representative CSSL	Positive allele
**PBN**	**qPBN1**	1	RM472	3.1	NS	0.051	-0.53	0.08	1.08	decreasing	38 918 631	35 483 127	37 890 275	34 691 456	L_10	*Og*
			RM165	15.78	*	0.2138	-1.0901	0.0002	3.70		41 165 563	37 610 087	40 107 107	36 797 582	L_46	*Og*
			RM104	9.94	NS	0.1531	-0.8265	0.003	2.58		41 226 405	37 667 912	40 167 993	36 855 255		
			RM3362	20.18	*	0.2581	-1.6642	3.42E-05	4.47		44 150 041	40 246 380	43 044 090	39 437 019		
	**qPBN2**	2	RM191	0.01	NS	0.0002	0.0510	0.91	0.04	decreasing	13 357 683	13 118 242	13 367 915	13 091 268	L_10	*Og*
			RM341	13.73	*	0.1968	-1.1670	0.0005	3.31		19 822 576	19 710 485	19 342 082	19 060 676	L_46	*Og*
			RM262	6.99	NS	0.1093	-0.6946	0.01	1.98		21 260 857	21 173 898	20 800 911	20 552 457		
	**qPBN7**	7	RM10	7.96	NS	0.1245	-0.9459	0.007	2.18	decreasing	22 227 559	20 124 022	22 189 175	19 801 671	L_10	*Og*
			RM351	14.76	*	0.2028	-1.4753	0.0003	3.52		23 991 049	21 742 091	23 926 002	21 416 312	L_46	*Og*
			RM234	6.35	NS	0.1019	-0.8551	0.015	1.84		25 545 695	23 016 334	25 473 749	22 717 218		
**PBL**	**qPBL1**	1	RM315	4.13	NS	0.07	0.50	0.05	1.33	increasing	37 748 125	34 409 891	36 735 198	33 646 637	L_10	*Og*
			RM472	12.82	*	0.18	0.71	0.0007	3.15		38 918 631	35 483 127	37 890 275	34 691 456	L_46	*Og*
			RM165	23.61	*	0.29	0.90	9.33E-06	5.03		41 165 563	37 610 087	40 107 107	36 797 582		
			RM104	14.83	*	0.21	0.69	0.0003	3.51		41 226 406	37 667 913	40 167 994	36 855 255		
			RM3362	11.95	*	0.17	0.96	0.001	2.99		44 150 041	40 246 380	43 044 090	39 436 568		
	**qPBL3**	3	RM55	0.61	NS	0.01	0.15	0.44	0.36	increasing	30 677 384	28 677 802	29 052 298	28 518 740	L_10	*Og*
			RM3525	12.67	*	0.18	0.55	0.0008	3.12		32 028 705	30 028 933	30 393 958	29 873 318	L_46	*Og*
			RM7000	11.89	*	0.18	0.44	0.001	2.97		35 524 810	33 277 548	33 802 689	33 128 938		
			RM227	0.99	NS	0.02	-0.21	0.324	0.49		36 679 424	34 296 048	34 932 380	34 146 855		
	**qPBL7**	7	RM10	8.52	NS	0.13	0.69	0.01	2.30	increasing	22 227 559	20 124 022	22 189 175	19 801 671	L_10	*Og*
			RM351	16.36	*	0.22	1.09	0.0002	3.81		23 991 049	21 742 091	23 926 002	21 416 312	L_46	*Og*
			RM234	9.21	NS	0.14	0.72	0.004	2.44		25 545 695	23 016 334	25 473 749	22 717 218		
**SBN/PBN**	**qSBN/PBN11**	11	RM21	0.002	NS	0.00	-0.02	0.90	0.05	decreasing	20 161 293	19 279 722	19 639 289	18 598 655	L_42	*Os*
			RM206	10.12	*	0.15	-0.37	0.002	2.63		22 961 364	21 790 549	22 480 895	21 092 821	L_55	*Og*
			RM254	12.14	*	0.18	-0.38	0.001	3.02		24 651 269	23 182 998	24 230 511	22 477 748	L_56	*Og*
			RM224	8.4	NS	0.13	-0.30	0.01	2.27		29 246 818	25 784 047	27 673 312	25 499 087		
	**qSBN/PBN12**	12	RM28130	8.78	NS	0.13	-0.49	0.004	2.35	decreasing	16 565 178	15 215 344	16 706 323	15 275 476	L_42	*Og*
			RM277	14.24	*	0.20	-0.43	0.0004	3.41		21 403 653	20 209 650	22 364 793	20 270 371	L_55	*-*
			RM235	2.5	NS	0.05	-0.21	0.12	0.92		25 324 296	23 473 458	26 141 561	23 604 232	L_56	*-*
**SpN**	**qSpN3**	3	RM60	10.13	*	0.15	-11.67	0.002	2.63	decreasing	109 164	86 928	106 972	77 908	L_55	*Og*
			RM22	8.98	NS	0.14	-10.52	0.004	2.39		1 561 917	1 493 255	1 520 630	1 485 562	L_11	*Og*
															L_26	*Os*
	**qSpN11**	11	RM21	0.55	NS	0.01	-5.72	0.46	0.34	decreasing	20 161 293	19 279 722	19 639 289	18 598 655	L_55	*Og*
			RM206	13.24	*	0.19	-22.90	0.001	3.23		22 961 364	21 790 549	22 480 895	21 092 821	L_11	*Os*
			RM254	13.61	*	0.19	-23.16	0.001	3.30		24 651 269	23 182 998	24 230 511	22 477 748	L_26	*Og*
			RM224	8.57	NS	0.13	-17.22	0.005	2.31		29 246 818	25 784 047	27 673 312	25 499 087		
**TBN**	**qTBN1**	**1**	RM226	0.31	NS	0.01	-0.06	0.58	0.23	increasing	35 105 017	31 957 160	34 034 101	31 200 058	L_10	*Og*
			RM265	22.66	*	0.28	0.21	1.33E-05	4.88		36 243 983	33 029 536	35 197 671	32 275 011	L_4	*Og*
			RM315	6.09	NS	0.10	0.16	0.02	1.78		37 748 125	34 409 891	36 735 198	33 646 637	L_46	
			RM472	31.11	*	0.35	0.26	6.70E-07	6.17		38 918 631	35 483 127	37 890 275	34 691 456		
			RM165	4.62	NS	0.07	0.12	0.04	1.45		41 165 563	37 610 087	40 107 107	36 797 582		
	**qTBN3-1**	**3**	RM175	1.93	NS	0.03	0.05	0.17	0.77	increasing	3 911 091	3 633 683	3 866 833	3 625 881	L_10	*Og*
			RM7576	21.91	*	0.27	0.23	1.76E-05	4.75		6 107 599	5 772 156	6 079 319	5 767 071	L_4	*Og*
			RM7	0.27	NS	0.00	-0.04	0.60	0.22		9 810 142	9 300 611	9 829 641	9 280 539	L_46	
	**qTBN3-2**	**3**	RM227	0.59	NS	0.01	-0.04	0.45	0.35	increasing	36 679 424	34 296 048	34 932 380	34 146 855	L_10	*Og*
			RM148	15.58	*	0.21	0.15	2.16E-04	3.67		37 552 877	35 129 500	35 843 050	34 980 430	L_4	*Og*
			RM85	0.72	NS	0.01	0.04	0.40	0.40		38 025 225	35 613 693	36 348 204	35 464 999		
	**qTBN5**	**5**	RM159	6.75	NS	0.11	0.13	0.01	1.92	increasing	496 697	433 400	488 155	404 817	L_4	*Og*
			RM267	25.12	*	0.30	0.47	5.38E-06	5.27		2 871 237	2 517 535	2 881 410	2 485 558		*Os*
			RM194	25.12	*	0.30	0.47	5.38E-06	5.27		5 179 705	4 653 443	5 329 987	4 472 981		
			RM169	0.96	NS	0.02	-0.06	0.33	0.48		7 361 060	6 805 643	7 498 060	6 637 671		
	**qTBN7**	**7**	RM11	0.00	NS	0.00	0.00	1.00	0.00	increasing	19 315 836	17 468 875	19 257 970	17 138 872	L_10	*Og*
			RM10	12.61	*	0.18	0.22	7.86E-04	3.10		22 227 559	20 124 022	22 189 175	19 801 671	L_4	*Og*
			RM351	17.42	*	0.23	0.30	1.02E-04	3.99		23 991 049	21 742 091	23 926 002	21 416 312	L_46	
			RM234	61.00	**	0.52	0.36	1.59E-10	9.80		25 545 695	23 016 334	25 473 749	22 717 218		
			RM18	62.09	**	0.52	0.37	1.09E-10	9.96		25 725 478	23 182 832	25 653 583	22 884 015		
			RM134	26.55	*	0.31	0.22	3.22E-06	5.49		26 708 250	24 069 776	26 637 574	23 770 479		
			RM118	33.95	*	0.37	0.27	2.63E-07	6.58		26 708 384	24 069 876	26 637 674	23 770 550		
			RM420	0.009	NS	0.00	-0.03	0.77	0.11		29 538 599	26 687 759	29 432 340	26 361 122		

*RL trait*: 13 CSSLs showed significant changes in RL value in comparison to the *Os_Caiapó* recurrent parent (Additional file 1: Table S4). However, no QTLs were detected for this trait (Additional file 2: Figure S1a).

*SBN/PBN trait*: a total of 16 CSSLs showed a significant difference in secondary branch number per primary branch (SBN/PBN ratio) compared to the *Og_MG12* parent (Table S4). Two QTLs associated with a decreased SBN/PBN ratio were detected (qSBN/PBN11 and qSBN/PBN12, maximum F-test scores of 15.09 and of 14.94 respectively) (Table 2; Additional file 2: Figure S1b). The average SBN/PBN ratio values were 3.0 for *Os_Caiapó* and of 1.7 for *Og_MG12*, corresponding to a decrease of 42% in *Og_MG12* relative to *Os_Caiapó*. The CSSLs L_42, L_55 and L_56 showed a decrease of 44.7%, 42.4% and 36.3% respectively relative to the *Os_Caiapó* parent, suggesting a high effect of the *Og_MG12* introgression(s) in these lines (Additional file 1: Table S4). The phenotyping of these lines was repeated for a second year to confirm panicle trait variation (Additional file 2: Figure S3). The L_55 and L_56 lines contain an *Og_MG12* segment in chromosome 11, but with missing marker information at the position of qSBN/PBN12. In contrast, the CSSL L_42 displays only an *Og_MG12* segment in chromosome 12 (Additional file 2: Figure S3).

*SpN trait*: a total of 12 CSSLs showed a significant difference compared to the *Os_Caiapó* recurrent parent (Additional file 1: Table S3). Two QTLs (qSpN3 and qSpN11) were associated with a decreased SpN with maximum F-test scores of 10.8 and 15.1 respectively (Table 2; Additional file 2: Figure S1c). CSSLs L_55, L_11 and L_26 showed decreases of 37.9%, 32.3% and 30.8% respectively compared to the *Os_Caiapó* parent (Additional file 1: Table S4). Lines L_55 and L_26 contain *Og* segments in both chromosomes 3 and 11 in the positions of the detected QTLs (Additional file 2: Figure S3). Line L_11 contains only one *Og_MG12* introgression in chromosome 11, related to qSpN11 (Additional file 2: Figure S3).

*PBN trait*: a total of 13 CSSLs had significantly different PBN values compared to the *Os_Caiapó* recurrent parent (Additional file 1: Table S4). Three QTLs corresponding to a decreased PBN (qPBN1, qPBN2 and qPBN7) with maximum F-test scores of 12.56, 15.49 and 16.44 were detected on chromosomes 1, 2 and 7 respectively (Table 2; Additional file 2: Figure S1d). Average PBN values were 11.9 for *Os_Caiapó* and 8.2 for *Og_MG12* (Additional file 1: Table S4). The PBN values of two CSSLs (L_10 and L_46) differed significantly from the *Os_Caiapó* recurrent parent with a reduction of 24.1% and 20.9% respectively (Additional file 1: Table S4). These two CSSLs harbor similar *Og_MG12* segments in chromosomes 1, 2 and 7 (Additional file 2: Figure S3).

*PBL trait*: Fourteen CSSLs exhibited significant differences with the *Os_Caiapó* recurrent parent for the PBL trait. Three QTLs corresponding to an increased PBL (qPBL1, qPBL3 and qPBL7) with maximum F-test scores of 22.55, 14.51 and 15.78 were detected on chromosomes 1, 3 and 7 respectively (Table 2; Additional file 2: Figure S1e). Average PBL values were 11.1 for *Os_Caiapó* and 12.3 for *Og_MG12* (Table 1). Two CSSLs (L_10 and L_46) showed increased PBL values of 19% and 12.8% respectively in comparison to the *Os_Caiapó* recurrent parent and harbored similar *Og_MG12* segments in chromosomes 1, 3 and 7 (Additional file 1: Table S4; Additional file 2: Figure S3).

*TBN trait*: Surprisingly, several CSSLs showed a significantly increased TBN value in our field conditions (Additional file 1: Table S3) and five QTLs were detected: qTBN1, qTBN3-1, qTBN3-2, qTBN5 and qTBN7 with maximum F-test scores of 31.11, 21.91, 15.98, 25.12 and 66.09 respectively (Table 2; Additional file 2: Figure S1f). The L_10 and L_4 lines have similar *Og_MG12* segments in chromosomes 1, 3 and 7. Within chromosome 5, only the L_4 line contains a segment from the *Og_MG12* parent (Additional file 2: Figure S3). As the L_46 line contains otherwise similar *Og_MG12* introgressions in chromosome 1, 3 and 7, this CSSL was included in the second phenotyping campaign, in which it revealed the presence of tertiary branches on its panicles in contrast to L_4 (Additional file 2: Figure S3). Overall, these results support an association of *Og_MG12* introgressions with the presence of tertiary branches.

**Fig. 3:**
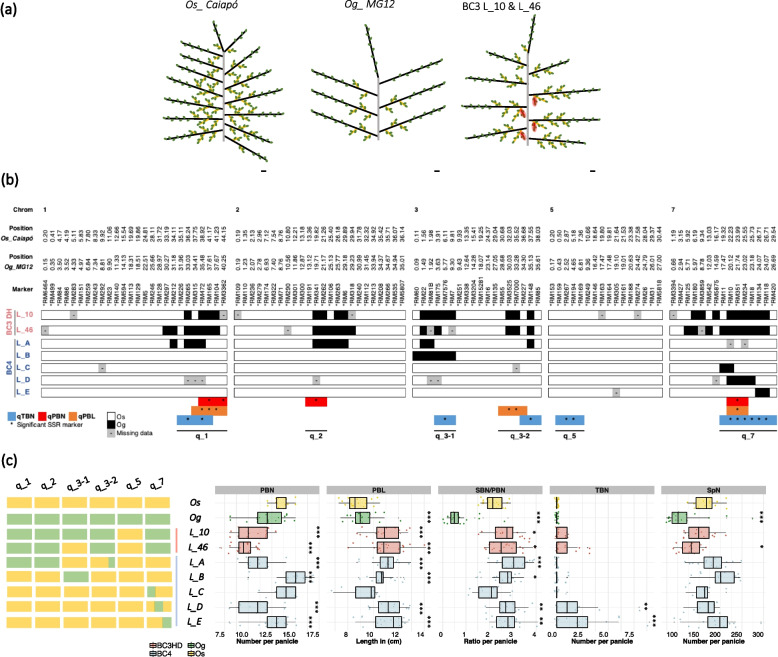
Phenotypic and genetic description of BC3DH and BC4 lines to dissect the effects of QTLs on panicle traits. **a** Schematic representation of the panicle structure observed in *Os_Caiapó*, *Og_MG12* and BC3DH L_10 and L_46, indicating primary branch (PB) in black, secondary branch (SB) in yellow, tertiary branch (TB) in red and spikelet (Sp) in green. Scale bar = 1cm. **b** Boxplots of the phenotypic variation observed in Os_*Caiapó* (yellow), *Og_MG12* (green), BC3HD (red) and BC4 (blue) lines. Each point represents the phenotypic value for one panicle. Statistical significance (*t*-test *p*-values) between *Os_Caiapó* and each line for the panicle morphological traits is indicated as follows: ** *p*-values < 0.01; *** < 0.001. The left-hand panel represents the allelic status (*Os_Caiapó* in yellow or *Og_MG12* in green) for each QTL region in each line. c Visualization of *Og_MG12* segments present in chromosomes 1, 3, 5 and 7 in BC3DH and BC4 lines to dissect the effects of QTLs on panicle traits. The positions of QTLs associated with PBN, PBL and TBN traits are indicated immediately above them (bars shaded in orange, red and blue respectively). Abbreviations: PBN, primary branch number; PBL, primary branch length; TBN, tertiary branch number; SBN/PBN, ratio between secondary branch and primary branch numbers; SpN, spikelet number

For the PBN, PBL and TBN panicle traits, the same BC_3_DH CSSLs (L_10 and L_46) led to the detection of several QTLs. The two lines in question display a similar panicle phenotype with the presence of tertiary branches associated with a decreased PBN and increased PBL and SBN/PBN ratio values compared to the *Os_Caiapó* recurrent parent (Fig. 3a). The L_10 and L_46 BC_3_DH lines contain a complex association of *Og_MG12* introgressions in their genomes (Fig. 3b). For clarification, the regions corresponding to colocalized QTLs, associated with different traits, were renamed without specifying the associated traits: q_1 (for qPBL1, qPBN1 and qTBN1); q_2 (for qPBN2), q_3-1 (for qTBN3-1); q_3-2 (for qPBL3 and qTBN3-2); q_5 (for qTBN5); and q_7 (for qTBN7, qPBN7 and qPBL7).

### Substitutions of *Os_Caiapó* genomic regions by corresponding *Og_MG12* segments can result in an added value panicle phenotype

As the altered phenotype observed in lines L_10 and L_46 was associated with multiple substitution segments (Fig. 3b), it was critical to determine whether this phenotype, and notably the formation of tertiary branches, involved one or several QTLs. Thus, different BC_4_F_3:5_ lines were obtained from the BC_3_DH lines by backcrossing and self-pollination (Fig. 3b), after which homozygosity was checked using the SSR markers carried by the introgressed *Og_MG12* segments present in the BC_3_DHs. Panicle traits were phenotyped for five BC_4_F_3_:_5_ lines (namely L_A to L_E) containing the different individual *O. glaberrima* introgressed segments affecting TBN, together with *Os_Caiapó* and *Og_MG12* (Fig. 3c; Additional file 1: Table S1).

Divergent panicle traits compared to the *Os_Caiapó* parent were observed in all BC_4_ lines except for L_C (Fig. 3b). The latter contains an introgression at the beginning of q_7 (from RM11 to RM10), suggesting that this region does not influence panicle architecture. All the other BC_4_ lines showed longer primary branches compared to the *Os_Caiapó* parent, suggesting that introgressions in chromosomes 1, 3 and 7 could independently influence primary branch length (Fig. 3c). The L_A line, containing *Og_MG12* regions corresponding to q_1, q_2 and q_3-2, additionally showed a decreased PBN value associated with a higher SBN/PBN ratio. This line did not produce any tertiary panicle branch. This suggests that the association of the q_1, q_2 and q_3-2 regions could cause a reduction in PBN associated with an increased SBN/PBN ratio.

Line L_B, containing only one *Og_MG12* introgression (from RM60 to RM7) in place of q_3-1, showed increased values for four panicle traits (PBN, PBL, SBN/PBN and SpN) but not for TBN. However, these results were not observed during the second phenotyping of this line, except for an increase in PBL (Additional file 2: Figure S4). Thus, it can be deduced that the q_3-1 region positively influences primary branch length in a manner that is independent of the other detected QTLs and that additive panicle trait effects may be observed depending on the environment.

Finally, the BC_4_ lines L_D and L_E were the only ones observed to develop tertiary branches (Fig. 3c). In both lines, this phenotype is associated with an increased SBN/PBN and a longer PBL. The higher SBN/PBN ratio is associated with a decreased PBN in L_D, meaning that in this line more secondary branch meristems are established during panicle development. Similar results for SBN/PBN and TBN were observed in a second round of phenotyping, with a comparable decrease in PBN in the L_E line (Additional file 2: Figure S4).

**Fig. 4:**
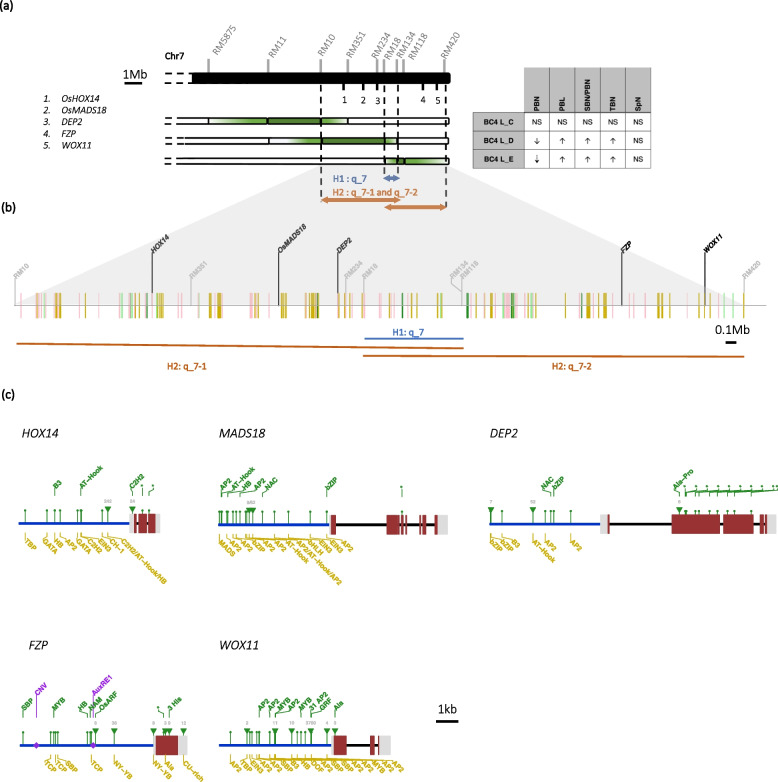
Description of genetic variations observed in the q_7 region between the *Os_Caiapó* and *Og_MG12* genomes (**a**) Influence of the q_7 region on panicle variation in the BC_4_ lines. Light green and green shading indicate the “extended” and strict introgression positions respectively for each BC4 line. **b** Representation of variation observed in the annotated genes between the *Os_Caiapó* and *Og_MG12* genomes in the region between the RM10 and RM420 SSR markers. Yellow bars show loci only present in *Os_Caiapó* genome, green bars indicate loci only present in *Og_MG12*. Light green bars represent genes that are duplicated in *Og_MG12* and pink bars represent genes that are annotated in *Og_MG12* but present in a different genomic region in *Os_Caiapó*. **c** Schematic representation of sequence divergence in candidate genes between the two genomes. Blue lines correspond to promoter regions, red and grey boxes represent exons and introns respectively. SNP and InDel variations leading to TFBS or amino acid changes are represented by dots and triangles respectively. The number above a triangle indicates InDel size. Asterisks indicate amino acid identity modification in the protein sequence. TFBSs present only in *Os_Caiapó* or *Og_MG12* are shown colored in yellow and green respectively. TFBS: Transcription Factor Binding Site

The two aforementioned lines contain contiguous introgression fragments in chromosome 7, from RM10 to RM18 for L_D and from RM134 to RM118 for L_E. Since the exact recombination positions of the introgressed segments for each BC_4_ is not known, the regions between SSRs with different genotypes are included in the QTL intervals. In Fig. 4a, it can be observed that lines L_D and L_E, which share the same phenotypes except for PBN, may harbor introgressions that overlap over only a small region between RM18 and RM134. Based on these observations, two different hypotheses can be proposed with regard to QTL position(s) in the q_7 region (Fig. 4a). The first one postulates the existence of a common QTL located between RM18 and RM134 that controls PBL, SBN/PBN and TBN. The second hypothesis is that two different QTLs, q_7-1 between RM10 and RM134, and q_7-2 between RM18 and RM420, exert a similar effect on PBL, SBN/PBN and TBN but act differently on PBN.

Taken together, the above results suggest that panicle architecture is a complex trait controlled by various different genomic regions which positively or negatively influence branching. An association of q_1, q_2 and q_3-2 produces opposing effects on PBN and SBN while only the q_3-1 region, comprised between RM175 and RM7, may impact positively upon PB length. Finally, the region in q_7, which negatively influences the number of primary branches and may be associated with the formation of tertiary branches and an increase in SBN per primary branch, may be defined as being either between the RM18 and RM134 marker positions or associated with two QTLs positioned between RM10 and RM134 for q_7-1 and from RM18 to RM420 for q_7-2.

### Co-location of QTLs with known genes and with QTLs detected in other populations

Many studies have reported QTLs associated with panicle architecture and/or yield traits or both, using bi-parental (QTL mapping) or diversity panels GWAS [25, 35, 41–49]. We found 76 common sites between the QTLs detected in this study and those reported earlier (Additional file 1: Table S5). Among them, five sites had been previously described in the evaluation of a CSSL population between *O. sativa* and *O. glaberrima* with respect to traits affecting panicle structure and grain yield [41]. In addition, co-located sites corresponding to similar traits (PBN, SBN and SpN) were found on chromosomes 11 and 12 through studies of various different rice populations [35, 43–45, 49]. Although the common sites identified all relate in some way to panicle architecture or yield, care should be taken when attempting to extrapolate between the different studies due to variations in the methodologies used to record traits.

To evaluate the synteny of the QTL regions between the genomes of the two CSSL parents, sequences from the assemblies of the *Os_Caiapó* and *Og_MG12* genomes obtained by ONT sequencing (https://doi.org/10.23708/QMM2WH; https://doi.org/10.23708/1JST4X; Additional file 1: Table S6) were used to carry out local alignment and structural variation analyses for each QTL region. The assembled genomes are of high continuity and completeness, with BUSCO score of 98.4% allowing precise detection of structural variations. For the majority of the QTL regions, no major structural variation was observed between the two genomes (Additional file 2: Figure S5). However, some notable differences were detected at the qPBN2, qTBN5, qTBN7, qSpN3 and qSBN12/PBN12 sites. Within QTLs qPBN2 and qTBN7, segments of respective lengths 1,296,899 and 230,762 bp in the *Os_Caiapó* genome were inverted in the *Og_MG12* genome (Additional file 1: Table S7). The genomic regions for QTLs qTBN5, qSpN3 and qSBN/PBN12 displayed large InDels between *Os_Caiapó* and *Og_MG12* (Additional file 1: Table S7). With the exception of qSBN11, we observed a high sequence similarity in the QTL regions between the two genomes, suggesting similar content in terms of coding sequences. Synteny analysis was extended by the alignment of the *Os_Caiapó* and *Og_MG12* QTL regions with those of the *O. sativa* cv. Nipponbare reference genome (Additional file 2: Figure S5). Based on the high conservation of the corresponding regions between *Os_Caiapó* and *Os_Nipponbare*, we then used as a reference, for subsequent analyses, the *O. sativa* cv. Nipponbare (IRGSPv1.0) functional gene annotation databases (i.e., MSU7 and RAP_db) for candidate gene prioritization in relation to panicle architecture and flowering.

For all QTLs identified in the present study, we found several known genes with relevant functions that related to flowering and/or panicle development (Additional file 1: Table S8; Additional file 2: Figure S6). Genes such as *LAX PANICLE1* (*LAX1*), *OsMADS1/LEAFY HULL STERILE1* (*LHS1*), *OsMADS14*, *OsMADS34/PANICLE PHYTOMER2* (*PAP2*)*, OsINDETERMINATE SPIKELET 1 (OsIDS1*), *OsMADS18*, *DENSE AND ERECT PANICLE2* (*DEP2*) and *FRIZZY PANICLE* (*FZP*) are known to be involved in the control of panicle development and were suggested as candidates that might contribute to the panicle branching diversity observed between the two parents *Os_Caiapó* and *Og_MG12* [3–5].

### q_7 genetic variations between *Os_Caiapó* and *Og_MG12*

We paid particular attention to the q_7 region, as it was found to be associated with several panicle morphological traits and to span a genomic region that included several panicle-associated genes. To test the hypothesis that specific genetic modifications present in the q_7 QTL region could be associated with variation in panicle architecture, we further compared the constituent genes in the region between the RM10 and RM420 markers in the *Os_Caiapó* and *Og_MG12* genomes on chromosome 7 (22.23 – 29.59 Mbp in *Os_Caiapó vs*. 20.12 – 26.69 Mbp in *Og_MG12*). For this purpose, gene annotation comparisons and BLAST analyses were performed to explore in detail gene synteny and presence/absence of genes between the *Os_Caiapó* and *Og_MG12* genomes within this region (Fig. 4b; Additional file 1: Table S11). Between the RM10 and RM420 marker positions, we observed a variation in the number of annotated genes between the two genomes (Additional file 1: Table S9). This variation is due to several factors: the differential presence of corresponding orthologs between the two genomes; differential gene duplication within the region; and different locations of a given gene within the two genomes (i.e., genomic rearrangement) (Fig. 4b; Additional file 1: Table S9). Among the genes that differ between the two genomes in this region, many are similar to transposable elements (TEs) or are hypothetical protein-encoding genes. None of them has a known function related to panicle development or control of flowering, or to any other developmental processes.

Special attention was paid to common genes present in the q_7 region that were known to be related to inflorescence development and/or meristem activity and maintenance. Based on a bibliographic survey, no candidate gene of special interest was identified within the region between the RM18 and RM134 markers. In contrast, several interesting candidate genes were identified in the q_7-1 and q_7-2 regions: the *OsHOX14*, *OsMADS18* and *DEP2* genes in q_7-1 and the *FZP* and *WOX11* genes in q_7-2 (Fig. 4c). SNP and InDel sites associated with the aforementioned genes that were polymorphic between the *Os_Caiapó* and *Og_MG12* genomes were analyzed in order to detect amino acid modifications, open reading frame alterations or transcription factor binding site (TFBS) variations within promoter regions.

*OsHOX14* (LOC_Os07g39320/Os07g0581700) encodes a protein of the Homeodomain-leucine zipper (HD-Zip) TF family [50, 51] and two of the identified SNPs cause amino acid variations in the coding sequence, including one in the homeobox domain (Fig. 4c; Additional file 1: Table S10). Comparison of the *OsHOX14* and *OgHOX14* promoter regions revealed InDel and SNP variations leading to the loss of nine TFBSs and a gain of three new TFBSs in the CSSLs bearing *Og_MG12* q_7-1 introgression (Fig. 4c; Additional file 1: Table S11).

*OsMADS18* (LOC_Os07g41370/Os07g0605200) encodes a protein of the AP1/FUL-like MADS-box TF subfamily [52, 53]. SNPs were detected in the coding sequence; one of the SNPs leads to a non-synonymous change in the OgMADS18 protein outside the known binding or functional domains (Fig. 4c; Additional file 1: Table S10). Scanning of the promoter regions of *OsMADS18* and *OgMADS18* in the *Os_Caiapó* and *Og_MG12* genomes respectively revealed several SNPs and InDels that lead to variations between the two genomes (presence/absence) of TFBSs recognized by AP2, NAC or HB TFs (Fig. 4c, Additional file 1: Table S11).

*DEP2* (LOC_Os07g42410/Os07g061600) encodes a plant-specific protein of unknown function [54]. Various SNPs and one InDel are observed between the coding sequences of *OsDEP2* and *OgDEP2* which result respectively in changed amino acid identities between the two protein orthologs (Fig. 4c; Table S9) and an insertion of two amino acids in the OgDEP2 protein. Sequence variations were also observed between the promoters of *OsDEP2* and *OgDEP2*; these variations lead to the loss of bZIP, B3, AT-Hook and AP2 TFBSs in the CSSLs carrying q_7. On the other hand, in comparison to *O. sativa*, the CSSLs carrying q_7 include NAC and bZIP TFBSs specific to the promoter of *OgDEP2*.

Among the genes contained in the q_7-2 region, *FZP* (LOC_Os07g47330/Os07g0669500) encodes an AP2/ERF TF [20, 55]. Several SNP and InDel variations observed in the coding region of *FZP* lead to coding sequence amino acid changes between the two genomes (Fig. 4c; Additional file 1: Table S9). An InDel of 9 bp in *OgFZP* results in the insertion of three histidine amino acids outside the single AP2 domain in the OgFZP protein. We observed several SNPs and InDels between the promoter of the two orthologs, which result in a differential presence of TFBSs (Fig. 4c, Additional file 1: Table S10). No variation was observed in the copy number variant (CNV) motif of 18 bp described by Bai et al. (2017) [18]. Huang et al. (2018) [19] revealed a deletion of 4 bp in the *OsFZP* 5' regulatory region in comparison to the sequence of *O. rufipogon*. This deletion is observed in *Os_Caiapó* and not in the genome of *Og_MG12* (Fig. 4c). Recently, it has been shown that CU-rich elements (CUREs) present in the 3' UTR of the *OsFZP* mRNA are crucial for efficient OsFZP translational repression [56]. Three CUREs are detected in the 3' UTR of *OsFZP* in the *Os_Caiapó* genome. In contrast, a deletion of the third CURE sequence is observed in the *Og_MG12* genome (Fig. 4c).

In the WUSCHEL-related homeobox *WOX11* gene (LOC_Os07g48560/Os07g0684900), an InDel in the coding region of *WOX11* leads to an Asn amino acid insertion in the OgWOX11 protein in *Og_MG12* without affecting the homeobox domain of the protein (Fig. 4c, Table S10). In the promoter region, variations between *Os_Caiapó* and *Og_MG12* lead to the presence/absence of several TFBSs in the promoter of *OgWOX11* (Fig. 4c; Table S11). We also observed a large insertion of 3,760 bp, containing about 31 AP2 TFBs, in *Og_MG12* compared to the *Os_Caiapó* genome.

Overall, our analysis revealed variations in synteny that demonstrate the absence of certain *Os_Caiapó* genes and the addition of other loci as a consequence of *Og_MG12* genomic introgressions within the CSSLs: some of these changes can be hypothesized to play a role in determining panicle trait diversity. Moreover, our analysis of candidate genes revealed TFBS variations and protein coding sequence polymorphisms that may lead to variations in transcript levels and/or protein activity in CSSLs harboring the q_7 introgression.

## Discussion

### Added value of the *O. sativa* x *O. glaberrima* CSSL population

Interspecific *O. sativa* x *O. glaberrima* CSSL genetic resources are of great interest: *O. glaberrima* provides a gene pool with high potential for rice improvement in terms of resistance to biotic and abiotic stresses and ecological adaptability [40, 41]. CSSL libraries are also good pre-breeding materials for the simultaneous identification, transfer and pyramiding of key genes in crop improvement programs. Genetic effects resulting in changes to panicle architecture could potentially be obtained via genome editing as an alternative to pyramiding QTLS producing the same effect. They are also useful for the study of traits lost or retained during the process of evolution, domestication and breeding [38, 57]. Genetic strategies based on CSSL populations have the advantage of using a relatively small number of lines for experiments, allowing replicating evaluations, thereby enhancing statistical strength for complex and time-consuming phenotypic assessments.

In this study, backcross introgression lines (BC_3_DH) of *O. sativa_Caiapó* x *O. glaberrima_MG12* were phenotyped for panicle morphological traits, which are key components of yield potential. Our analyses led to the detection of 15 QTLs, localized on chromosomes 1, 2, 3, 5 and 7 and comprising several genes known to be involved in panicle architecture determination (Table 2). Colocalization was observed with previous QTLs and GWAS sites associated with panicle branching traits, supporting the implication of the QTLs detected in this study in the regulation of panicle morphology. Moreover, since the genomic sites mentioned above were detected using different sets of populations, we propose that the genomic regions in question play a major role in the determination of panicle architecture diversity.

For most of the traits studied, we detected several QTLs within the same line, as observed in the lines L_10 and L_46 that allowed the localization of QTLs governing TBN-, PBN- and PBL-related traits. This is not surprising in the case of a complex trait such as panicle architecture, which is controlled by a large number of genes and QTLs with small effects that can be influenced by environmental and epistatic interactions [58]. The fact that we observed a colocalization of QTLs for different panicle traits suggests a likely genetic interdependence between these traits; this hypothesis is supported by correlations observed between phenotypic values for morphological characters such as SpN, SBN, PBN, PBL and RL that share common QTLs.

### Impact of *O. glaberrima* introgressions on panicle traits in the *O. sativa* background

Compared with the *Os_Caiapó* recurrent parent, the *Og_MG12* donor parent displays a global reduction in the number of panicle constituents (i.e., numbers of primary branches, secondary branches and spikelets) and produces longer primary branches. Tertiary branches were not observed in the *Og_MG12* donor parent. On the basis of these observations, the QTLs detected in the CSSLs, as expected, were associated with a global decrease in panicle constituent numbers (PBN, SBN, SpN) and an increase of PB length in comparison to the *Os_Caiapó* parent. In most of the lines, introgressions of *Og_MG12* alleles of panicle-regulating genes produced a negative effect on the regulatory network controlling panicle morphological traits, indicating that some of the genomic variations observed between *Og_MG12* and *Os_Caiapó* in these regions are functionally important.

Surprisingly, we also detected QTLs associated with an increase in panicle trait values in comparison to the *Os_Caiapó* recurrent parent. For example, the *Og_MG12* introgression localized on chromosome 3 in place of q_3-1 induces a globally higher complexity of panicle branching with an increase in PBN, SBN/PBN and SpN, associated with an increased PBL. This region includes the gene *OsMADS1/LHS1* belonging to the *SEPALLATA MADS-Box* TF family, which is known to play an important role in determining floral meristem identity and in floral organ development [59–61]. In *O. sativa*, *OsMADS1/LHS1* directly regulates several other transcription factor genes (MADS box and Homeodomain family members) and hormone signaling pathways implicated in floral meristem specification, maintenance and determinacy [62]. These unexpected effects indicate that introgression of certain *Og_MG12* regions into the *Os_Caiapó* genetic background can lead to an enhanced panicle phenotype and suggest a perturbation of the native pathways regulating inflorescence development in *Os_Caiapó*. This perturbation could be explained by several mechanisms. Firstly, it is possible to postulate the presence of a negative regulator of branching in *Os_Caiapó* that is absent in the *Og_MG12* introgression, leading to an upregulation of branching in the CSSL. A second hypothesis could be proposed whereby one or more genes specific to *O*. *glaberrima* are present in the *Og_MG12* introgression compared to the corresponding *Os_Caiapó* region. One or more of these genes could be positive regulators of panicle architecture, with this regulatory potential being either latent or only very weakly expressed in the *Og_MG12* background. A third possible explanation could be proposed, involving *cis*-variations associated with orthologous genes conserved between the two genomes. In this case, the addition of new *Og_MG12* genes, or their variant allelic forms into the *Os_Caiapó* genomic background could be postulated to exert an additive effect on panicle branching in the CSSLs, by affecting regulatory interactions within the *Os_Caiapó* network governing panicle branching.

In a similar way, our observations on the effects of QTL q_7, which favored increased SB and TB numbers, might have more than one possible explanation. Comparative analysis of the BC_4_ lines led us to suggest two hypotheses (above) regarding the position of QTL(s) in the q_7 region (Fig. 4a). However, based on quantitative and qualitative differences between the BC_4_ line phenotypes, we favor the hypothesis whereby two distinct regions influence panicle trait variation differentially (i.e., the q_7-1 and q_7-2 regions). In this scenario, both would be associated with increased PBL, SBN and TBN traits values whereas q_7-1 would have an additional negative effect on PBN. Moreover, the overlapping region of the introgressed segments in BC_4_ lines L_D and L_E, which spans a region of ~ 982 Kbp, is recombined in both lines. This implies that lines L_D and L_E can bear the *Os* allele at any locus in this region, so the probability that both lines bear the *Og* allele at a same putative QTL position is low. In addition, this region only contains a few genes whose predicted function is unknown.

In parallel, we observed a good synteny between the two species within the q_7-1 and q_7-2 regions, ruling out the possibility that large-scale genomic rearrangements might explain the phenotypic variations observed. Indeed, the strong global synteny between these regions suggests rather that CSSL phenotypes might be accounted for by modifications within the genomic regions of key regulatory genes that give rise to variations in their expression or in their protein functions and/or interactions through gene regulatory networks (GRNs). Mutations affecting transcription factor proteins and/or DNA sequences or both, such as enhancers and promoters, fall into this category [63]. Moreover, traits associated with dynamic processes are more readily modified through their *cis*- and/or *trans*-regulation rather than through coding sequence mutations [64]. The q_7-1 and q_7-2 regions comprise several genes known for their role in panicle development and numerous SNPs and InDels have been detected in the promoter regions and coding sequences of these candidate genes.

Within the q_7-1 region, three genes have been reported to have a direct function in panicle architecture establishment. The homeodomain-leucine zipper transcription factor gene *OsHOX14* has been shown to be involved in the regulation of panicle development and its loss of function leads to a reduction in PBN compared to wild type [50, 51]. The second candidate gene *OsMADS18*, in addition to its role in flowering time promotion [53], is known to specify inflorescence meristem identity through interaction with PANICLE PHYTOMER2 (PAP2/OsMADS34) and the two other members of the AP1/FUL subfamily, OsMADS14 and OsMADS15 [14]. *DEP2* encodes a plant-specific protein without a known functional domain, and some of its mutations affect elongation of the rachis and both primary and secondary branches, caused by a defect in cell proliferation [54, 65, 66]. For each of these three genes, the *Og_MG12* allelic version shows variations in comparison to *Os_Caiapó* in both protein coding sequences and in putative TFBSs within the promoter region. The presence of these binding sites may reveal a divergence in the regulation of the expression of these genes between the two parents, which could lead to the morphological variations observed in the CSSLs (i.e., decrease in PBN).

Concerning the q_7-2 region associated with the formation of higher order panicle branches (i.e., secondary and tertiary branches), the candidate genes *WOX11* and *FZP* were detected in both genomes. The WUSCHEL-related homeobox gene *OsWOX11* is necessary and sufficient to promote crown root emergence and growth in rice by either responding to or by regulating auxin and cytokinin signaling [67–72]. Recently, Cheng et al. (2018) [73] showed that OsWOX11 and JMJ705 cooperatively control shoot growth and commonly regulate the expression of a set of genes involved in meristem identity, indicating that the role of this gene is not confined to roots. A comparison of the promoter regions of *WOX11* orthologs revealed numerous SNP and InDel differences between the two genomes and an insertion of 3,760 bp in *Og_MG12*, which includes numerous AP2 TFBS potentially recognized by the *MULTI-FLORET SPIKELET 1 (MFS1)* and *OsFZP* genes, known to play an important role in the regulation of spikelet meristem determinacy [12, 20, 55, 74]. In an *O. sativa* genomic environment, the promoter of *OgWOX11* containing new AP2 TFBSs could potentially be programmed differently in its expression during the reproductive phase of the plant due to altered promoter activity, with possibly effects on cell proliferation in the axillary meristem leading to higher order branching in the CSSL lines.

The *OsFZP* gene plays an important role in the transition from branch to spikelet primordium during panicle development [12, 20, 55]. Reduced expression of *OsFZP* at the reproductive stage increases the extent of higher order branching of the panicle, resulting in increased grain number [24]. Recent studies showed that fine tuning of *OsFZP* expression at the transcriptional and post transcriptional levels could affect panicle architecture [18, 19, 21, 56, 75]. Some SNPs and InDel variations between the *Os_Caiapó* and *Og_MG12* genomes were detected within the regulatory regions of the respective *FZP* orthologs, with notable polymorphic sites occurring close to an auxin response element (AuxRE) 2.7 Kpb upstream of *OsFZP* and within the CU rich elements (CURES) localized in the 3'UTR of the same gene [19, 56]. These two variations observed in regulatory regions of *OgFZP* could result in an increase in transcript and/or protein amounts, leading to a precocious transition from branch to spikelet primordium. Such a scenario would be corroborated by the lesser-branched panicle phenotype of *Og_MG12* compared to *Os_Caiapó* but not by the phenotype of the CSSLs containing *OgFZP*. The analysis of the combinatorial effect of variations in these regulatory elements would be useful to explain the observed phenotypes.

## Conclusion

This study was carried out with the broad aim of identifying discrete genetic elements that govern rice panicle architectural diversity using a population of interspecific introgression lines. We detected several QTLs associated with rice yield and panicle branching diversity observed between the two cultivated rice species *O. sativa* and *O.* *glaberrima*, with decreased values – as expected – in the CSSLs but also unexpectedly with added values for some QTLs compared to the *O. sativa* recurrent parent. This was the case for QTL q_7 on chromosome 7, which causes an increase in numbers of higher order panicle branches attributable to two distinct genomic regions: q_7-1 and q_7-2. A detailed comparative genomic analysis revealed variations in gene content, promoter region sequence and protein coding sequences for a number of different key genes that act in the control of panicle architecture.

When an *Og_MG12* allelic variant is placed in an *Os_Caiapó* genomic background context within a CSSL, it might in some instances cause increased panicle branching by acting in an additive fashion, through enhancement of the native *Os_Caiapó* gene regulatory network (GRN) that governs panicle development. Importantly, the biological functions and regulation of a number of developmentally important genes present in the q_7-1 and q_7-2 regions have already been described in *O. sativa*; however, functional analyses of their *O. glaberrima* orthologs have yet to be undertaken in order to confirm the conservation of biological roles and/or to assess their regulation. Further studies assessing how the genes of interest are regulated will be beneficial to the improvement of rice breeding strategies for the exploitation of favorable alleles present in this CSSL population. The genetic material created here will provide knowledge and resources to facilitate the retention of favorable alleles in breeding programs and to develop new improved rice cultivars using *O. glaberrima* as a genetic resource for enhancing morphological traits in *O. sativa*.

## Materials and methods

### Plant materials

The plant materials consisted in a set of 60 interspecific introgression lines, or Chromosome Segment Substitution Lines (CSSL). The CSSLs were derived at CIAT, Cali, Colombia from a cross between *O. sativa* subgroup tropical japonica (cv. Caiapó) as the recurrent parent and *O. glaberrima* (cv. MG12; acc. IRGC103544) as the donor parent [40]. The CSSLs used were BC_3_DH, that is, obtained after three rounds of backcrossing to the recurrent parent followed by double haploidization of BC_3_F_1_ male gametes (Additional file 1: Table S1). They were genotyped at CIAT using 200 simple-sequence repeat (SSR) markers [40]. The program CSSL Finder v1.0.0 (http://mapdisto.free.fr) [40] was then used to select the 60 lines from an initial panel of 312 BC_3_DH lines, so that the entire donor genome was represented by overlapping chromosome segments. The effects of *O. glaberrima* introgressions on panicle trait variation were evaluated in five BC_4_F_3:5_ lines, derived so that they contained the target region and as few introgressed *O. glaberrima* genomic segments in the rest of the genome as possible (Additional file 1: Table S1).

### Phenotypic evaluation

The 60 BC_3_DH lines, along with the parents (*Os*_*Caiapó* and *Og*_*MG12*), were grown together in November 2012-January 2013 (Year 1) in the International Center for Tropical Agriculture (CIAT, now Alliance Bioversity-CIAT) headquarters experimental fields (Palmira, Colombia) (76° 21'W, 3° 30'N and 967masl) under irrigated conditions. The experimental design was a randomized complete block design with two replications and 62 plots. Five plants per line per plot were grown. Panicle traits were evaluated for subsequent QTL analysis and validation. For each line, the three main panicles from three randomly chosen plants (9 panicles total) per line per replicate were collected. Each panicle was spread out on a white background and held in place with metal pins for photography shooting. A total of 1,443 panicles from the 60 BC_3_DH lines and the parents were dissected and scored manually. In January-April 2014 (Year 2), a total of nine BC_3_DH lines were re-evaluated using P-TRAP software [76], which performs automatic scoring of panicle traits. Five BC_4_F_3_/F_5_ lines with parents, plus L_10 and L_46 BC_3_DH, were grown in the greenhouse in Montpellier, France (March 2021) at 28 °C / 80% relative humidity under short days conditions (11h light-13h dark), and were scored for panicle traits using P-TRAP. Panicle phenotypes were re-evaluated in the same conditions for three BC_4_ lines (L_B, L_D and L_E in June 2021).

For each analysis (2013, 2014 and 2021), 6 morphological panicle traits were scored: rachis length (RL); number of primary branches (PBN); number of secondary branches (SBN); number of tertiary branches (TBN); spikelet number (SpN); and average primary branch length (PBL) per panicle (Additional file 1: Table S2).

### Statistical analysis and QTL detection

To check for genotypic and genotype $\times$ environment effects, analysis of variance (ANOVA) was performed on each trait in the R software package (version 4.0.4, R Core Team 2022), taking into consideration replicates and lines as fixed effects:

$${y}_{ij}=\mu +{g}_{i}+{r}_{j}+{\varepsilon }_{ij}$$

where ${y}_{ij}$ is the phenotype score of the line $i$ in repetition $j$, $\mu$ is the average of phenotypic values across the population, ${g}_{i}$ is the random effect of genotype $i$, ${r}_{j}$ is the random effect of repetition $j$ and ${e}_{ij}$ is a residual error associated with line $i$ and repetition $j$. The ANOVA results showed that lines differed significantly for all traits (*p*-value $<0.001$) (Additional file 1: Table S3), indicating strong genotypic effects. The factor "repetition" was also significant for the RL, PBL and traits, although it was much less significant than the genotype effect (Additional file 1: Table S3). Thus, we decided to carry a preliminary QTL analysis – with the methodology described hereby – on each repetition separately. As a result, the F-test profiles were much similar between the two repetitions (Additional file 2: Figure S1). Therefore, the subsequent analyses presented in this work were done taking the average phenotypic values of the two repetitions.

Trait heritability was computed using the line effect based on the variance among phenotypic measurements between the two replicates of the population phenotyping assay. The corrplot (function cor), ade4 (function dudi.pca using center = TRUE and Scale = TRUE as arguments) and devtool R packages were used to analyze phenotypic correlation between traits.

The CSSL Finder program was used to display graphical genotypes of the 200 SSRs in the 60 BC_3_DH CSSLs together with phenotypic values for each trait. Putative QTLs were detected using the graphical genotyping tool proposed by CSSL Finder, which allows combination of logical genotype–phenotype association with single-marker ANOVA1 F-test. The F-test was considered significant when its value was higher than 10.0, which corresponds to a $p-value<\sim 0.002$ considering the degrees of freedom of our experimental design.

Because the F-test can lead to detection of false QTLs when the numbers of lines with and without an introgression in the considered region, putative QTLs were submitted to a comparison between the CSSLs and the recurrent parent *Os*_*Caiapó* was performed by a Dunnett's multiple comparison test ($p<0.05$) (glht function in R). The relative effect *RE* of an *O. glaberrima* introgression in a given CSSL was calculated using the least square means (LSMEANS) output of the GLM procedure as follows:

$$RE=100\times \left[LsMean\left(Lines\right)-LSMean\left(Os\_Caiap{\acute{o} }\right)\right]/LSMean\left(Os\_Caiap{\acute{o} }\right)$$

A putative QTL detected by the F-test was considered as validated when the trait values of the lines bearing the introgression at the QTL location were significantly different—according to the Dunnett's test—from that of the parent *Os_Caiapó* $(p<0.001)$ and when at least two lines bearing overlapping segments shared similar phenotypes. The phenotypic variation of these lines was further evaluated in the second phenotyping experiment in Year 2.

### Genome sequencing, assembly, quality assessment and annotation

Samples of young leaves from *O. sativa* cv. Caiapó and *O. glaberrima* cv. MG12 were used to extract genomic DNA and for preparation of SKL-LSK109 libraries as described in [77]. Genome sequencing was performed on a Nanopore MinION Flow Cell R9.4.1 (Oxford technologies, Oxford Science Park, UK). The long-read sequences of *Os_Caiapó* and *Og_MG12* generated by the ONT sequencing were assembled using the following steps. After basecalling and Q > 8 filtering with Guppy V6.1.2, with the SUP model, the preliminary genome was assembled with Flye V2.9 and default assembly parameters for Sup ONT [78]. Medaka V1.6.1 was then used to create consensus sequences and to improve the accuracy of the assembly (polishing). The contigs were ordered with RagTag V2.1.0 using *O.sativa* cv. Nipponbare*/IRGSP1.0* as a reference and with default parameters [79]. An evaluation of genome assembly completeness was carried out using BUSCO (Benchmark Universal Single Copy Orthologs) v5.2.2 with default parameters and the Poale database (Additional file 1: Table S7) [80].

The Liftoff tool was used for gene prediction and annotation using the genome reference *O. sativa* cv. Nipponbare (IRGSPv1.0, MSU7.0) and associated gff files for both *Os_Caiapó* and *Og_MG12* and by using the *O. glaberrima* genome reference OglaRS2 along with its associated gff files (https://ftp.gramene.org/oryza/release-6/gff3/oryza_glaberrima/) in the case of *Og_MG12* (Additional file 1: Table S7) [81].

### Genomic alignment

The sequences of the QTL regions and the genes of interest were extracted from the three genomes *O. sativa* cv. Caiapó, *O. glaberrima* cv. MG12 and *O. sativa* cv. Nipponbare MSU7 using Seqkit version 2.4.0 [82]. Sequences of QTL regions were aligned and plotted using the minimap2 aligner implemented in the D-GENIES online tool (https://dgenies.toulouse.inra.fr/run) using the option Few repeats.

### QTL colocalization, identification and bio-analysis of candidate genes

For QTL and gene colocalization, the qTARO database (http://qtaro.abr.affrc.go.jp), the funRiceGenes database (https://funricegenes.github.io/) [83] and various datasets from recently published works were used to identify QTLs and genes that overlapped with the QTL regions detected in this study. To identify genes potentially associated with the QTL regions, we used the *O. sativa* cv. Nipponbare MSU7.0 (https://riceplantbiology.msu.edu) and the RAP_db (https://rapdb.dna.affrc.go.jp) annotation databases. From the annotated gene list, the candidate genes were identified based on their predicted function (biological processes), their referencing in the funRiceGenes database and/or their expression pattern with respect to the trait of interest [34, 84]. Regions spanning the candidate genes and their associated 5 Kbp upstream region (considered as their promoter regions) were obtained for *O. sativa* (IRGSPv1.0, MSU7.0) and for *O. glaberrima* (OglaRS2), then nucleotide and protein alignments were performed using CLUSTALW in order to identify SNPs, InDels and amino acid changes between the two species [85]. The Plant Promoter Analysis Navigator web facility (http://plantpan.itps.ncku.edu.tw/) was used to detect transcription factor binding sites (TFBSs) and regulatory elements (CpG islands and tandem repeats), with a cut-off at 0.8, in the promoter region of candidate genes [86]. Variations between the two genomes, in terms of promoter and gene structures along with SNP and InDel positions and information, were drawn using the R package KaryoploteR [87].

### Supplementary Information

**Additional file 1:** **Table S1.** CSSL information showing *Og_MG12* introgression(s) for each line and their localization(s) (chromosome and left-right SSR markers). Target fragments were those selected so as to constitute the whole *Og_MG12* genome with the 60 CSSLs. **Table S2.** Quantification of panicle traits in the CSSLs and parents *Os_Caiapó *and O.g_MG12. Abbreviations: RL, Rachis Length; PBN, Primary Branch Number; PBL, Primary Branch Length; SBN, Secondary Branch Number; SpN, Spikelet Number; SBN/PBN, ratio of SBN and PBN. **Table S3.** Variance analysis done on panicle traits for the 60 CSSLs and their parents phenotyped the year 1. **Table S4.** Significant line*trait associations observed in the population studied based on a Dunnett's test. **Table S5.** QTL and GWAS sites colocalized with the QTLs detected in this study. **Table S6.** Metrics of genome quality obtained for *Os_Caiapó* and *Og_MG12* genome ONT sequencing assemblies. **Table S7.** Summary of the positions (in bp) of structural variations observed in the QTLs detected. **Table S8.** Genes described in the FunRiceGenes database located within each QTL detected in this study. **Table S9.** Gene synteny and description of loci annotated between the RM10 and RM420 markers in *Os_Caiapó* (RAP_db and MSU annotations) and *Og_MG12* (RAP_db, MSU and OglaRS2). Genes highlighted in yellow are absent in the q_7 region of *Og_MG12*, genes highlighted in green are annotated in the *Og_MG12* q_7 region but are absent in the Os_Caiapó genome, genes highlighted in light green are duplicated in the *Og_MG12* q_7 region and genes highlighted in pink are annotated in the *Og_MG12* q_7 region, are absent in the *Os_ Caiapó* q_7 region but are present in other chromosomes in the *Os_Caiapó* genome. **Table S10.** Amino acid modifications in proteins encoded by candidate genes in q_7. **Table S11.** TFBS variations in the promoters of candidate genes present in q_7. Abbreviations: EIN3, ETHYLENE-INSENSITIVE-LIKE3; C2H2, C2H2-Type Zinc finger; AP2, AP2-ERF; bHLH, basic helix-loop-helix; TCP, TEOSINTE-BRANCHED1; SBN, SQUAMOSA BINDING PROTEIN; NY-YB, nuclear factor Y; HB, HOMEODOMAIN; MADS, MADS-BOX; bZIP, basic leucine zipper; TBP, TATA-box-binding.

**Additional file 2:** **Figure S1.** CSSL Finder screenshots showing graphical representations of the genotypes of the 60 BC_3_DH lines along with corresponding data for each evaluated panicle trait across the two repetitions together and for each repetition separated (rep1 and rep2). (a) RL, Rachis Length; (b) SBN/PBN, Secondary Branch Number per Primary Branch; (c) SpN. Spikelet Number; (d) PBN, Primary Branch Number; (e) PBL, Primary Branch Length; (f) TBN, Tertiary Branch Number.. The 12 chromosomes are displayed vertically. They are covered by 200 evenly dispersed SSR markers. The genotypes of the individual lines are displayed horizontally. Shading indicates the allelic composition of chromosomes. Light gray areas represent the *Os_Caiapó* genetic background, black areas represent the *Og_MG12* chromosome segments, dark gray areas represent the heterozygous segments and blue areas correspond to missing data. On the right, solid color bars indicate the values of the panicle traits tested for each line. At the bottom of each graph, the dotted line indicates the statistical threshold of the F-test for the evaluated panicle trait. **Figure S2.** Graphic representation of the genotypes of the 60 BC3DH CSSLs, showing line x trait significant associations and QTL positions. The 12 chromosomes are covered by 200 evenly dispersed SSR markers. Genotypes are displayed horizontally. Black areas represent the *O. glaberrima* MG12 target chromosome segments, the set of segments broadly covering the entire *O. glaberrima* MG12 genome. White areas represent the *O. sativa* Caiapó genetic background and grey areas represent *O. glaberrima* additional chromosomal segments. Phenotypic effects of CSSL lines are represented in relative terms as circles on the right of the figure. The area of each circle is proportional to its relative effect. Horizontal bars at the bottom of the figure indicate QTL positions deduced from analyses performed using CSSL finder software. The colors of the circles and bars represent the effect as follows: red, increasing effect compared to the recurrent parent Os_Caiapó; green; decreasing effect. **Figure S3.** Genetic and phenotypic description of CSSLs showing the extreme significant differences for the panicle trait analyzed (A) SBN/PBN, (B) SpN, (C) PBN, (D) PBL and (E) TBN. Upper subpanel: Chromosome graphical representation of *Og_MG12* introgression positions in the CSSL. Position of the SSR markers is indicated in Mbp. Lower subpanel: boxplots of the phenotypic variation observed in Year 1 and Year 2 in these lines. Each point represents the phenotypic value for one panicle. Statistical significance (t-test *p*-values) between *Os_Caiapó* and each line for the panicle morphological trait is indicated as follows: NS if the test is non-significant; *p-values < 0.05; **<0.01; ***<0.001. Abbreviations: PBN, primary branch number; PBL, primary branch length; TBN, tertiary branch number; SBN/PBN, ratio between secondary branch and primary branch numbers; SpN, Spikelet number. **Figure S4.** Phenotypic description of BC_4_ lines to dissect the effects of QTLs on panicle traits. Boxplot of the phenotypic variation observed in Os_Caiapó (yellow), *Og_MG12* (green), and BC_4_ (blue) lines in greenhouse (2021). Each point represents the phenotypic value for one panicle. Statistical significance (t-test *p*-values) between Os_Caiapó and each line for the panicle morphological traits is indicated as follows: ** *p*-values <0.01; ***<0.001. Abbreviations: PBN, primary branch number; PBL, primary branch length; TBN, tertiary branch number; SBN/PBN, ratio between secondary branch and primary branch numbers; SpN, spikelet number. **Figure S5.** Dotplots of the minimap2 alignment (implemented in D-GENIES web facilities) of panicle trait-related QTLs detected between the *Os_Caiapó* and *Og_MG12* genomes and between each of the latter aligned against *O. sativa* cv. Nipponbare as a reference genome. QTL coordinates in each genome are indicated on axis X and Y. Dot colors are relative to the identity value (I) which is a BLAST-like alignment identity (I=Number of bases, including gaps per number of matching bases in the bases). **Figure S6.** Physical map positions of detected QTLs and colocalizations with known genes related to branching and flowering.

## Data Availability

The CSSLs materials are available from the corresponding authors on reasonable request. Sequencing data generated for this project are available at https://doi.org/10.23708/QMM2WH and https://doi.org/10.23708/1JST4X for *Os_Caiapó* and *Og_MG12* respectively.

## Abbreviations

RL: Rachis Length
PBN: Primary Branch Number
PBL: Primary Branch Length
SBN: Secondary Branch Number
SpN: Spikelet Number
SBN/PBN: Ratio of SBN per PBN
*Os_Caiapó*: *Oryza sativa* Cv*.* Caiapó;
*Og_MG12*: *Oryza glaberrima* Cv. MG12
CSSL: Chromosome Segment Substitution Lines
BC: Backcross
SRR: Simple Sequence Repeat
TFBS: Transcription Factor Binding Site
