# Supplementary material for: Genomic introgressions from African rice (Oryza glaberrima) in Asian rice (O. sativa) lead to the identification of key QTLs for panicle architecture
Source: BMC Genomics. 2023 Oct 4;24:587. doi: 10.1186/s12864-023-09695-6 (PMC10548634; doi:10.1186/s12864-023-09695-6)
Supplement: Supplementary file 2 — Additional file 2: Figure S1. CSSL Finder screenshots showing graphical representations of the genotypes of the 60 BC3DH lines along with corresponding data for each evaluated panicle trait across the two repetitions together and for each repetition separated (rep1 and rep2). (a) RL, Rachis Length; (b) SBN/PBN, Secondary Branch Number per Primary Branch; (c) SpN. Spikelet Number; (d) PBN, Primary Branch Number; (e) PBL, Primary Branch Length; (f) TBN, Tertiary Branch Number.. The 12 chromosomes are displayed vertically. They are covered by 200 evenly dispersed SSR markers. The genotypes of the individual lines are displayed horizontally. Shading indicates the allelic composition of chromosomes. Light gray areas represent the Os_Caiapó genetic background, black areas represent the Og_MG12 chromosome segments, dark gray areas represent the heterozygous segments and blue areas correspond to missing data. On the right, solid color bars indicate the values of the panicle traits tested for each line. At the bottom of each graph, the dotted line indicates the statistical threshold of the F-test for the evaluated panicle trait. Figure S2. Graphic representation of the genotypes of the 60 BC3DH CSSLs, showing line x trait significant associations and QTL positions. The 12 chromosomes are covered by 200 evenly dispersed SSR markers. Genotypes are displayed horizontally. Black areas represent the O. glaberrima MG12 target chromosome segments, the set of segments broadly covering the entire O. glaberrima MG12 genome. White areas represent the O. sativa Caiapó genetic background and grey areas represent O. glaberrima additional chromosomal segments. Phenotypic effects of CSSL lines are represented in relative terms as circles on the right of the figure. The area of each circle is proportional to its relative effect. Horizontal bars at the bottom of the figure indicate QTL positions deduced from analyses performed using CSSL finder software. The colors of the circles and bars rep [file 12864_2023_9695_MOESM2_ESM.pdf]

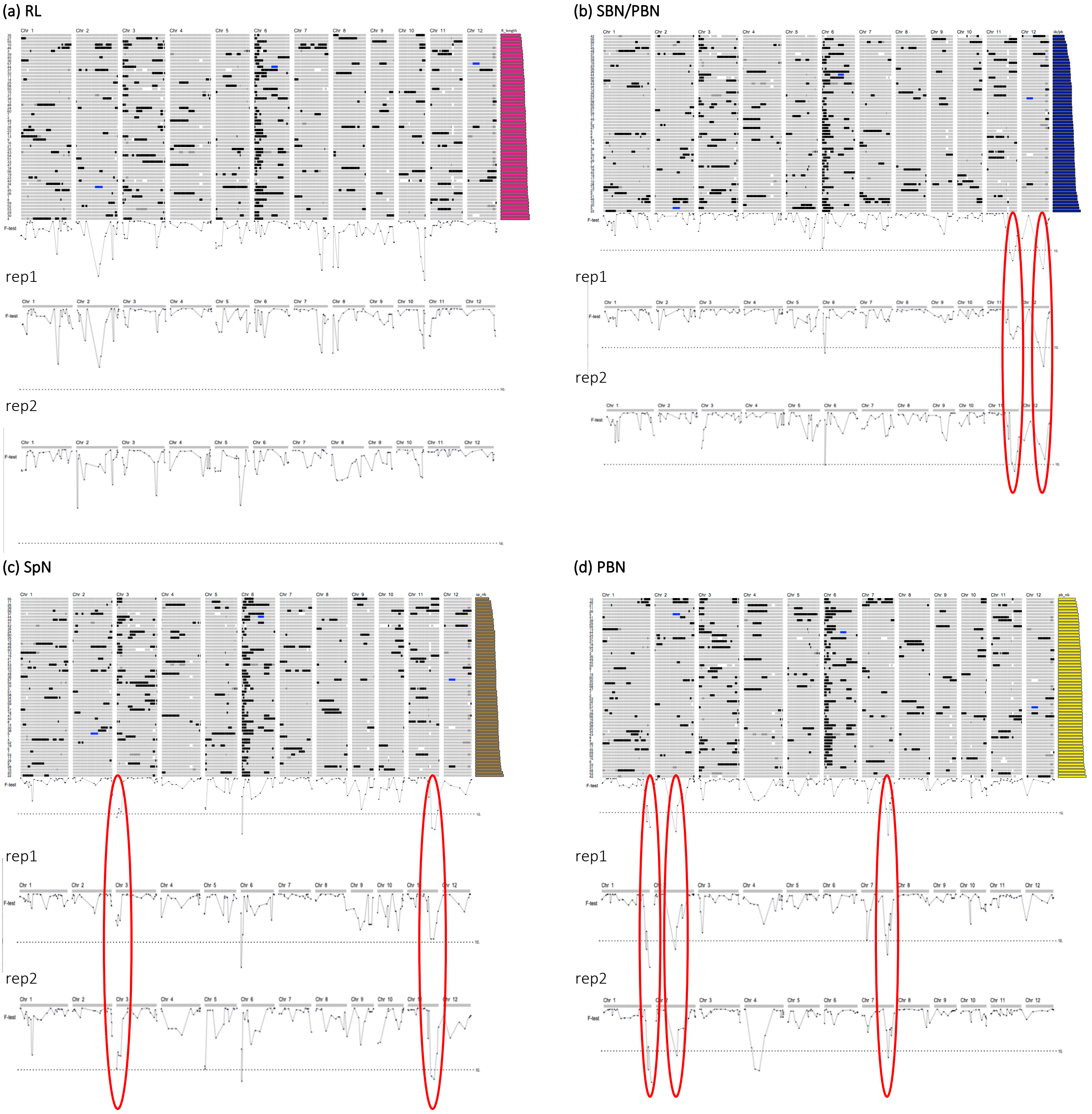

(a) RL
rep1
rep2
(b) SBN/PBN
rep1
rep2
(c) SpN
rep1
rep2
(d) PBN
rep1
rep2
Chr 1
Chr 2
Chr 3
Chr 4
Chr 5
Chr 6
Chr 7
Chr 8
Chr 9
Chr 10
Chr 11
Chr 12
F-test

(e) PBL

(f) TBN

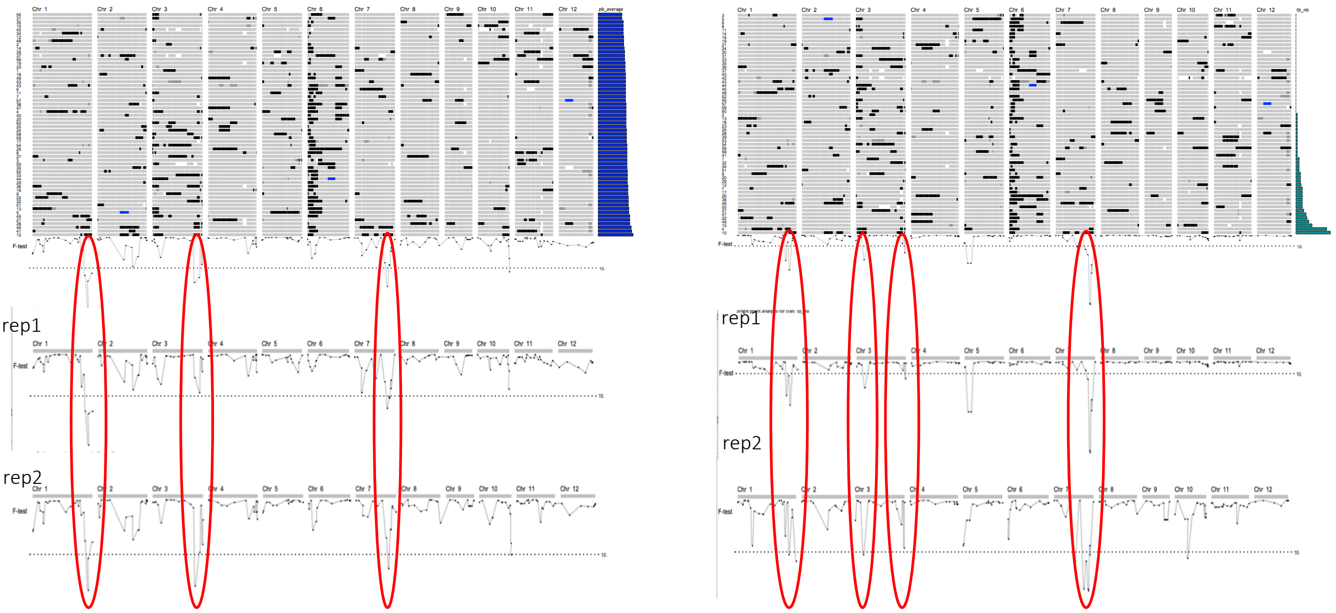

Additional file 2: Figure S1. CSSL Finder screenshots showing a graphical representations of the genotypes of the 60 BC_3DH lines along with corresponding data for each evaluated panicle trait across the two repetitions together and for each repetition separated (rep1 and rep2). **(a)** RL, Rachis Length; **(b)** SBN/PBN, Secondary Branch Number per Primary Branch; **(c)** SpN. Spikelet Number; **(d)** PBN, Primary Branch Number; **(e)** PBL, Primary Branch Length; **(f)** TBN, Tertiary Branch Number. The 12 chromosomes are displayed vertically. They are covered by 200 evenly dispersed SSR markers. The genotypes of the individual lines are displayed horizontally. Shading indicates the allelic composition of chromosomes. Light gray areas represent the *Os_Caiapó* genetic background, black areas represent the *Og_MG12* chromosome segments, dark gray areas represent the heterozygous segments and blue areas correspond to missing data. On the right, solid color bars indicate the values of the panicle traits tested for each line. At the bottom of each graph, the dotted line indicates the statistical threshold of the F-test for the evaluated panicle trait.

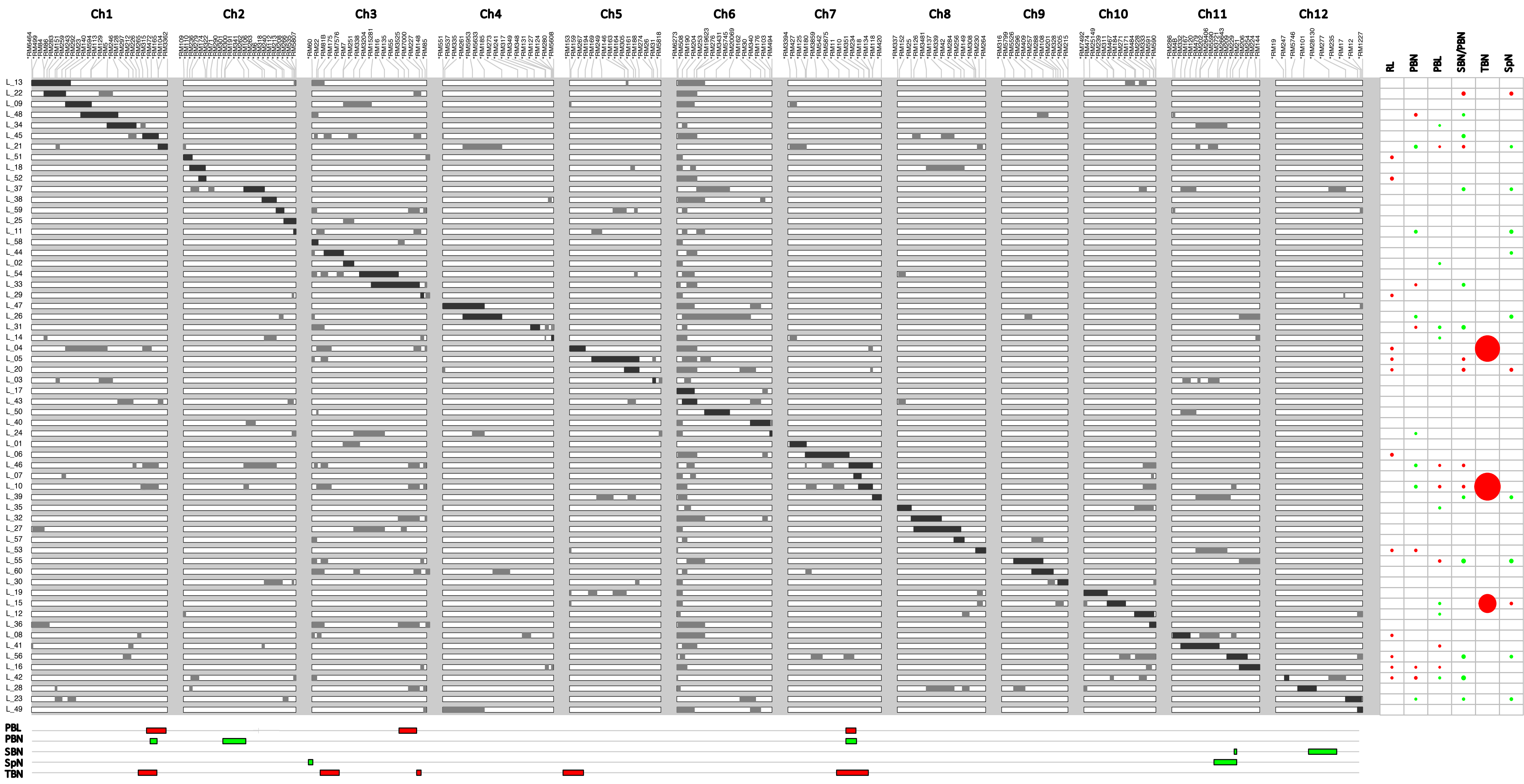

Additional file 2: Fig. S2 Graphic representation of the genotypes of the 60 BC3DH CSSLs, showing line x trait significant associations and QTL positions. The 12 chromosomes are covered by 200 evenly dispersed SSR markers. Genotypes are displayed horizontally. Black areas represent the *O. glaberrima* MG12 target chromosome segments, the set of segments broadly covering the entire *O. glaberrima* MG12 genome. White areas represent the *O. sativa Caiapó* genetic background and grey areas represent *O. glaberrima* additional chromosomal segments. Phenotypic effects of CSSL lines are represented in relative terms as circles on the right of the figure. The area of each circle is proportional to its relative effect. Horizontal bars at the bottom of the figure indicate QTL positions deduced from analyses performed using CSSL finder software. The colors of the circles and bars represent the effect as follows: red, increasing effect compared to the recurrent parent *Os_Caiapó*; green; decreasing effect.

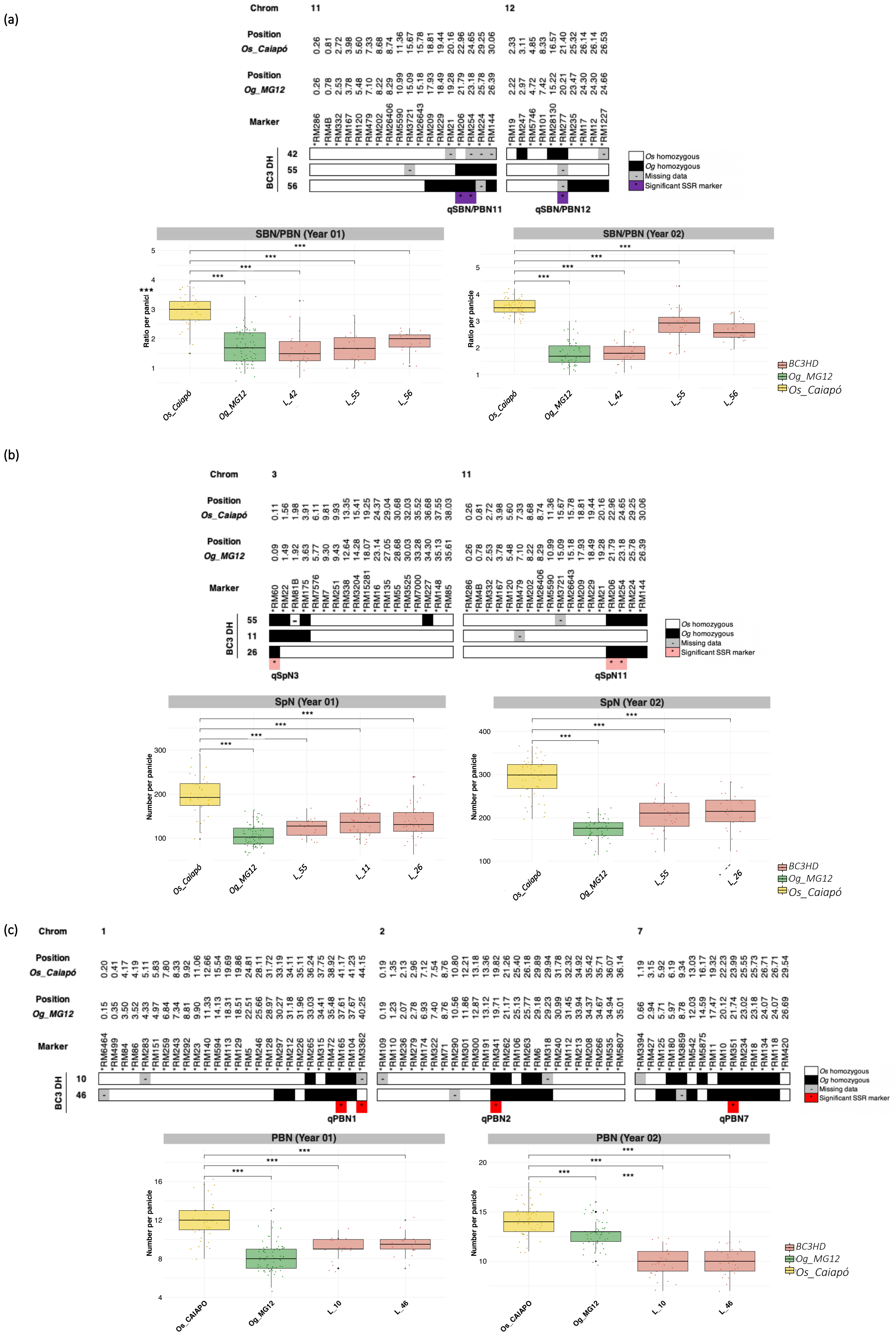
(a)
Chrom
11
12
Position
Os_Caiapó
Position
Og_MG12
Marker
BC3 DH
42
55
56
Os homozygous
Og homozygous
Missing data
Significant SSR marker
qSBN/PBN11
qSBN/PBN12
SBN/PBN (Year 01)
SBN/PBN (Year 02)
Ratio per panicle
Os_Caiapó
Og_MG12
L_42
L_55
L_56
BC3HD
Og_MG12
Os_Caiapó
(b)
Chrom
3
11
Position
Os_Caiapó
Position
Og_MG12
Marker
BC3 DH
55
11
26
Os homozygous
Og homozygous
Missing data
Significant SSR marker
qSpN3
qSpN11
SpN (Year 01)
SpN (Year 02)
Number per panicle
Os_Caiapó
Og_MG12
L_55
L_11
L_26
BC3HD
Og_MG12
Os_Caiapó
(c)
Chrom
1
2
7
Position
Os_Caiapó
Position
Og_MG12
Marker
BC3 DH
10
46
Os homozygous
Og homozygous
Missing data
Significant SSR marker
qPBN1
qPBN2
qPBN7
PBN (Year 01)
PBN (Year 02)
Number per panicle
Os_CAIAPO
Og_MG12
L_10
L_46
BC3HD
Og_MG12
Os_Caiapó

(d)

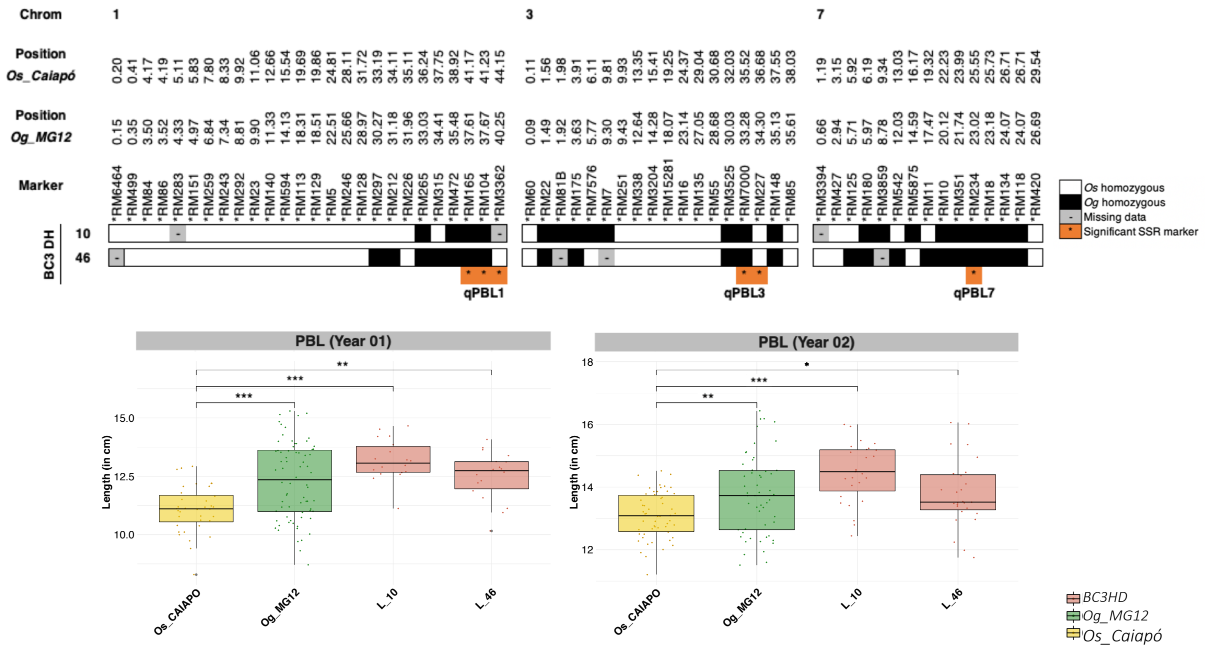

(e)

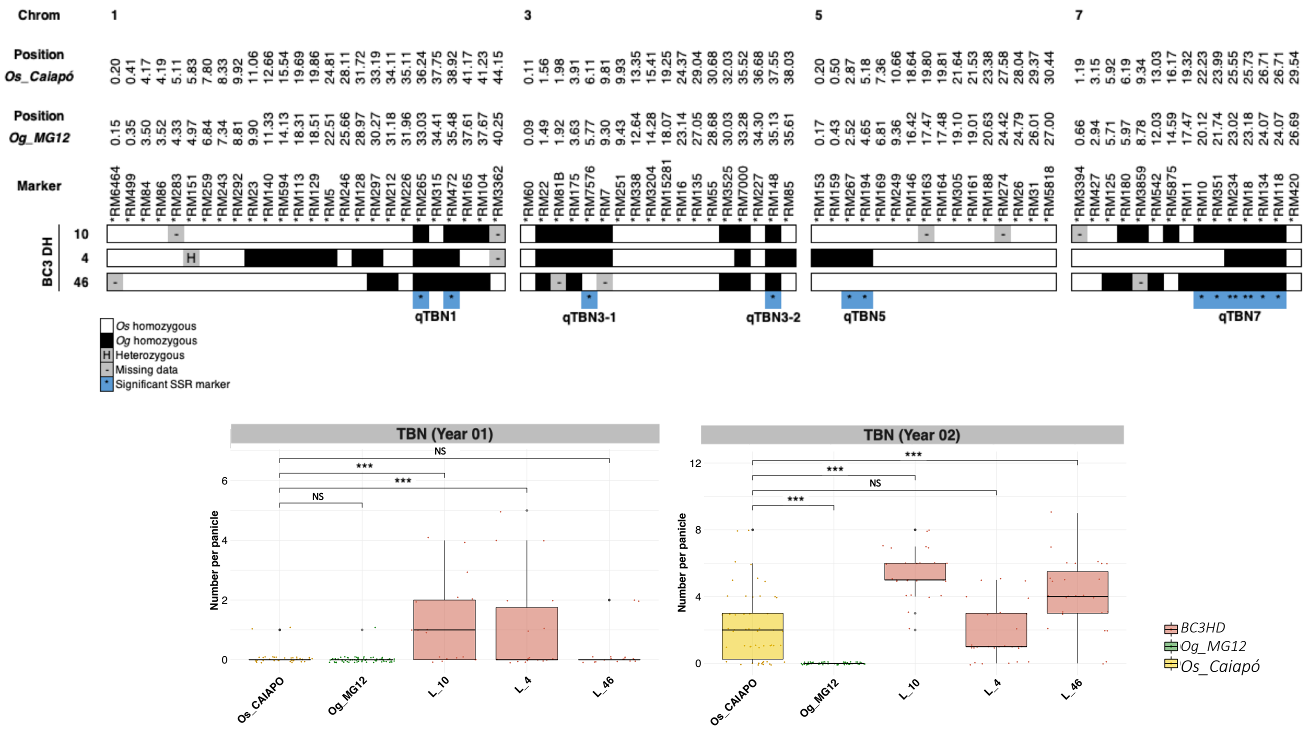

Additional file 2: Figure S3. Genetic and phenotypic description of CSSLs showing the extreme significant differences for the panicle trait analyzed **(a)** SBN/PBN; **(b)** SpN; **(c)** PBN; **(d)** PBL; and **(e)** TBN. Upper subpanel: Chromosome graphical representation of *Og_MG12* introgression positions in the CSSL. Position of the SSR markers is indicated in Mbp. Lower subpanel: boxplots of the phenotypic variation observed in Year 1 and Year 2 in these lines. Each point represents the phenotypic value for one panicle. Statistical significance (*Dunnett*-test *p*-values) between *Os_Caiapó* and each line for the panicle morphological trait is indicated as follows: NS if the test is non-significant; *p-values < 0.05; **<0.01; ***<0.001. Abbreviations: PBN, primary branch number; PBL, primary branch length; TBN, tertiary branch number; SBN/PBN, ratio between secondary branch and primary branch numbers; SpN, Spikelet number.

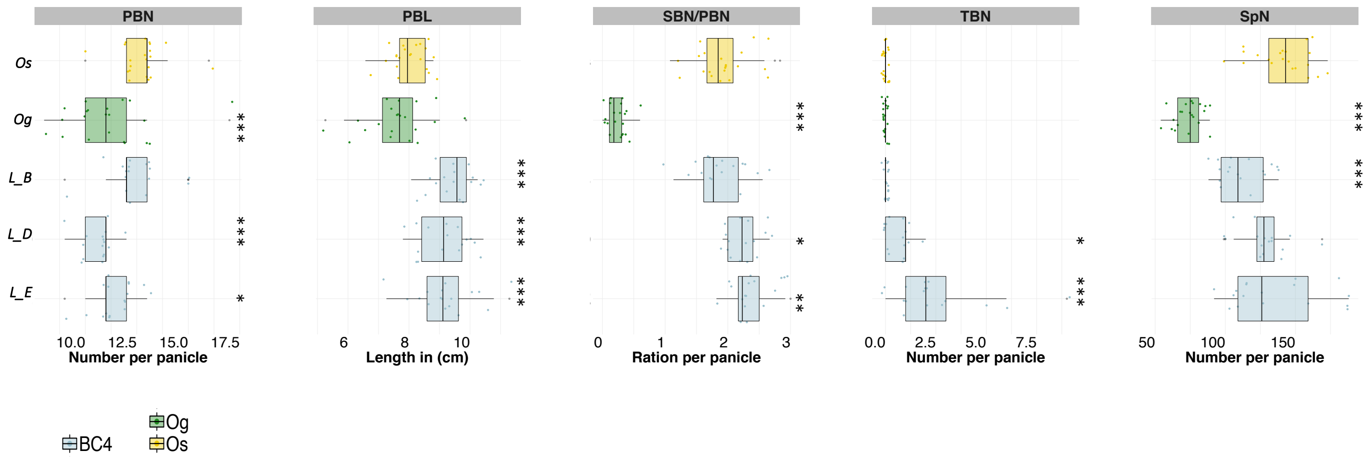

Additional file 2: Figure S4. Phenotypic description of BC_4 lines to dissect the effects of QTLs on panicle traits. Boxplot of the phenotypic variation observed in *Os_Caiapó* (yellow), *Og_MG12* (green), and BC_4 (blue) lines in greenhouse (2021). Each point represents the phenotypic value for one panicle. Statistical significance (*Dunett p*-values) between *Os_Caiapó* and each line for the panicle morphological traits is indicated as follows: *p-values < 0.05; **<0.01; ***<0.001. Abbreviations: PBN, primary branch number; PBL, primary branch length; TBN, tertiary branch number; SBN/PBN, ratio between secondary branch and primary branch numbers; SpN, spikelet number.

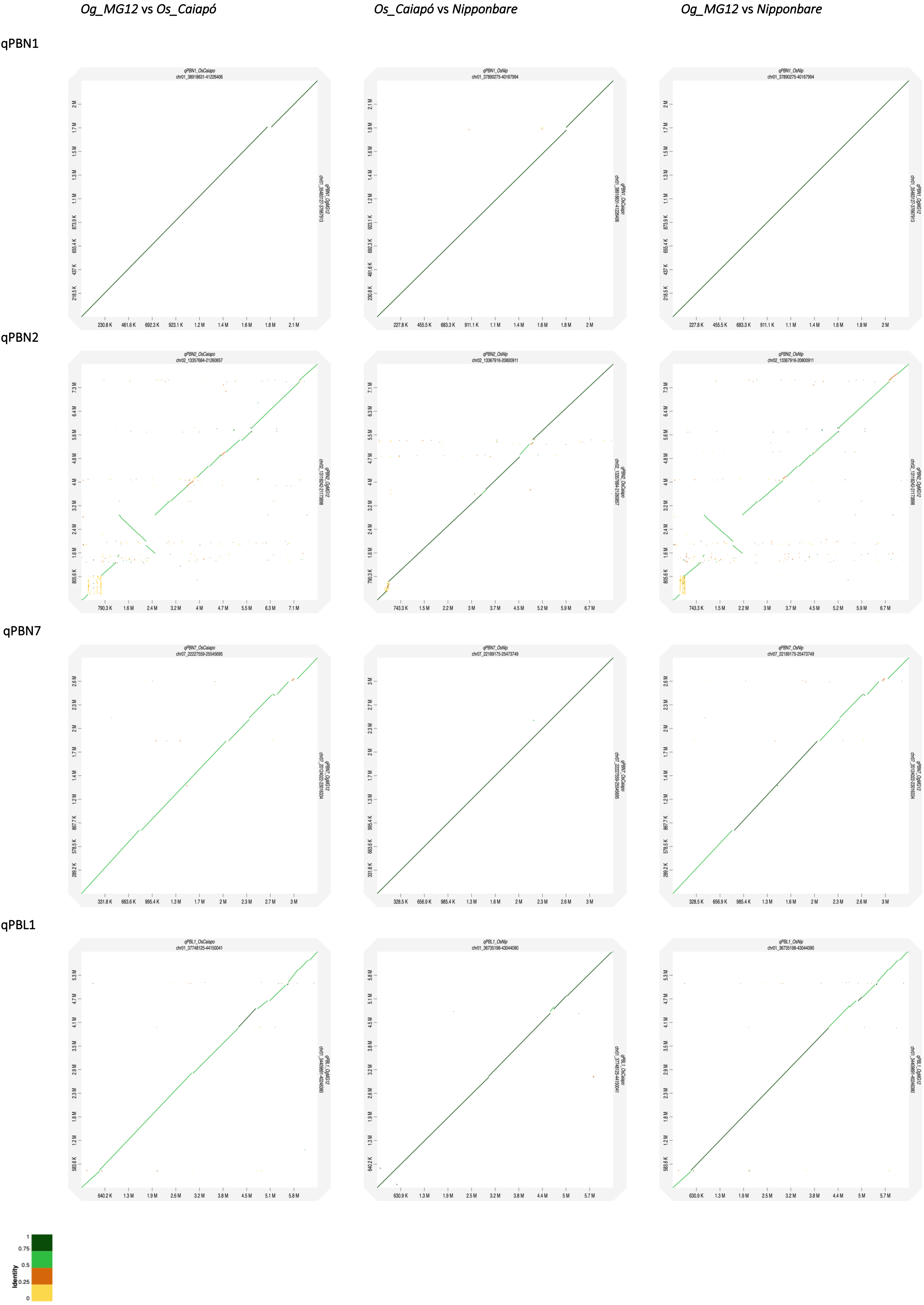
Og_MG12 vs Os_Caiapó
Os_Caiapó vs Nipponbare
Og_MG12 vs Nipponbare
qPBN1
qPBN2
qPBN7
qPBL1
Identity
1
0.75
0.5
0.25
0

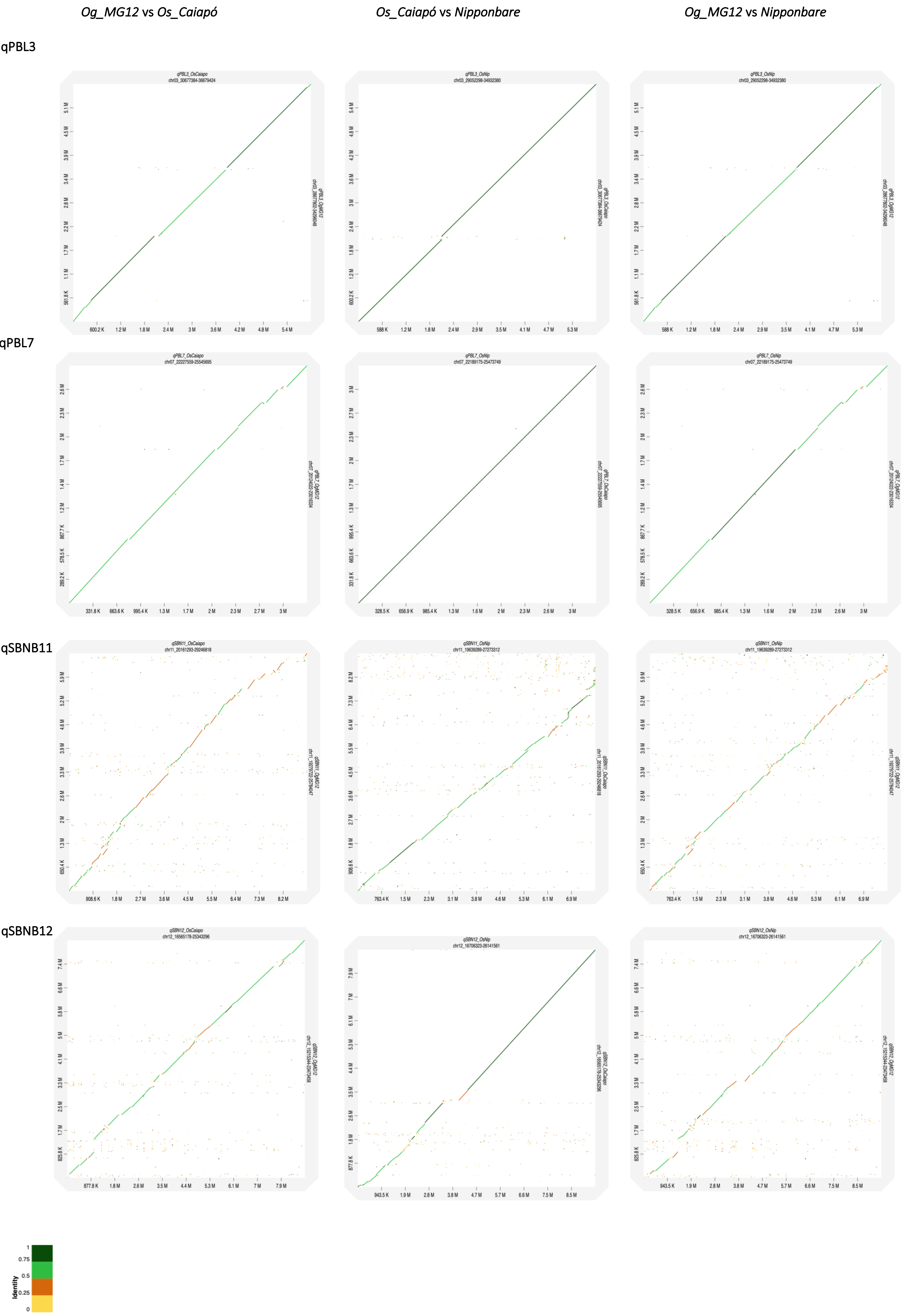
Og_MG12 vs Os_Caiapó
Os_Caiapó vs Nipponbare
Og_MG12 vs Nipponbare
qPBL3
qPBL7
qSBNB11
qSBNB12
Identity
1
0.75
0.5
0.25
0

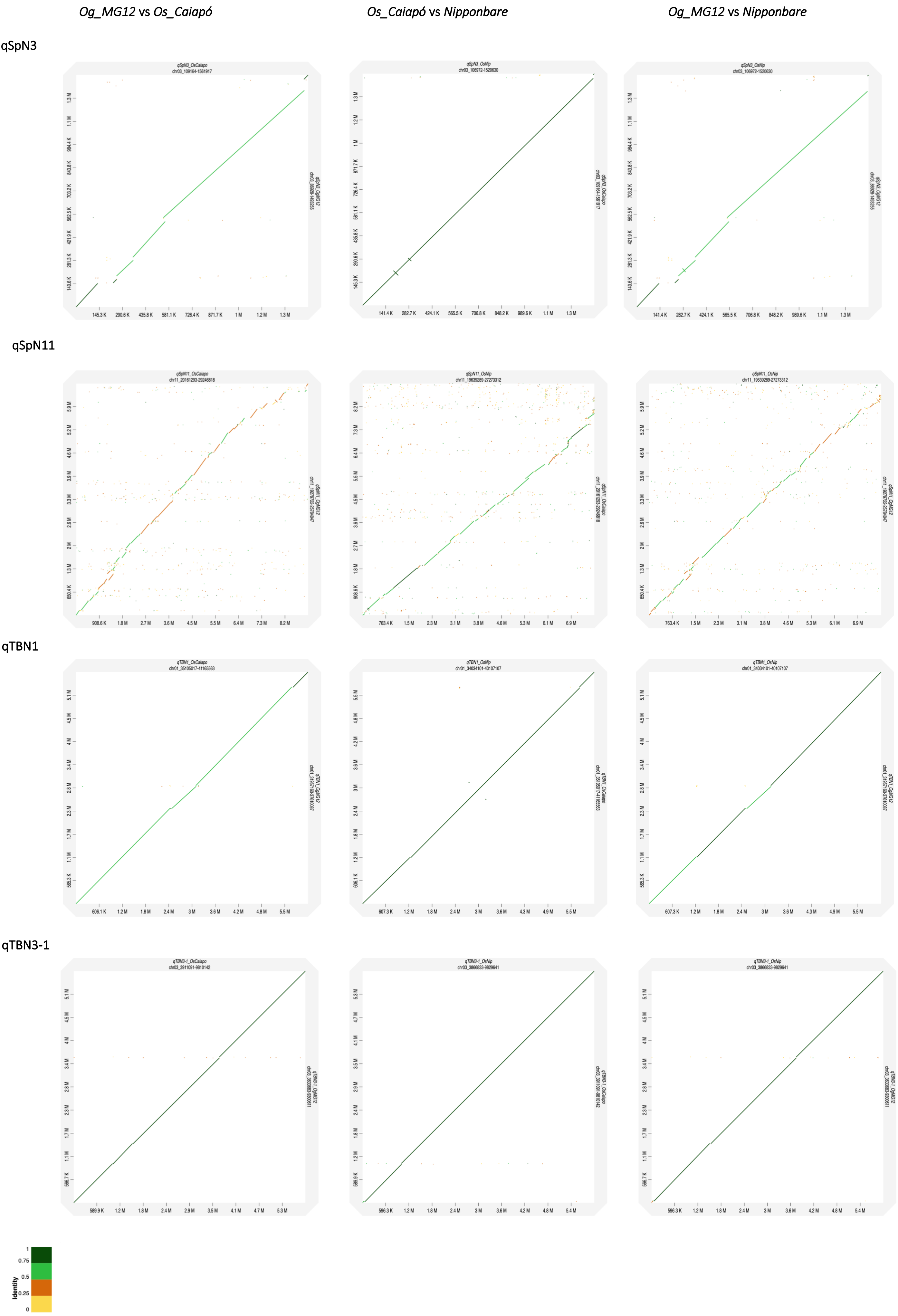

Og_MG12 vs Os_Caiapó
Os_Caiapó vs Nipponbare
Og_MG12 vs Nipponbare
qSpN3
qSpN3_OsCaiapo
chr03_109164-1561917
qSpN3_OsNip
chr03_106972-1520630
qSpN11
qSpN11_OsCaiapo
chr11_20161293-29246818
qSpN11_OsNip
chr11_19639289-27273312
qTBN1
qTBN1_OsCaiapo
chr01_35105017-41165563
qTBN1_OsNip
chr01_34034101-40107107
qTBN3-1
qTBN3-1_OsCaiapo
chr03_3911091-9810142
qTBN3-1_OsNip
chr03_3866833-9829641
Identity
1
0.75
0.5
0.25
0

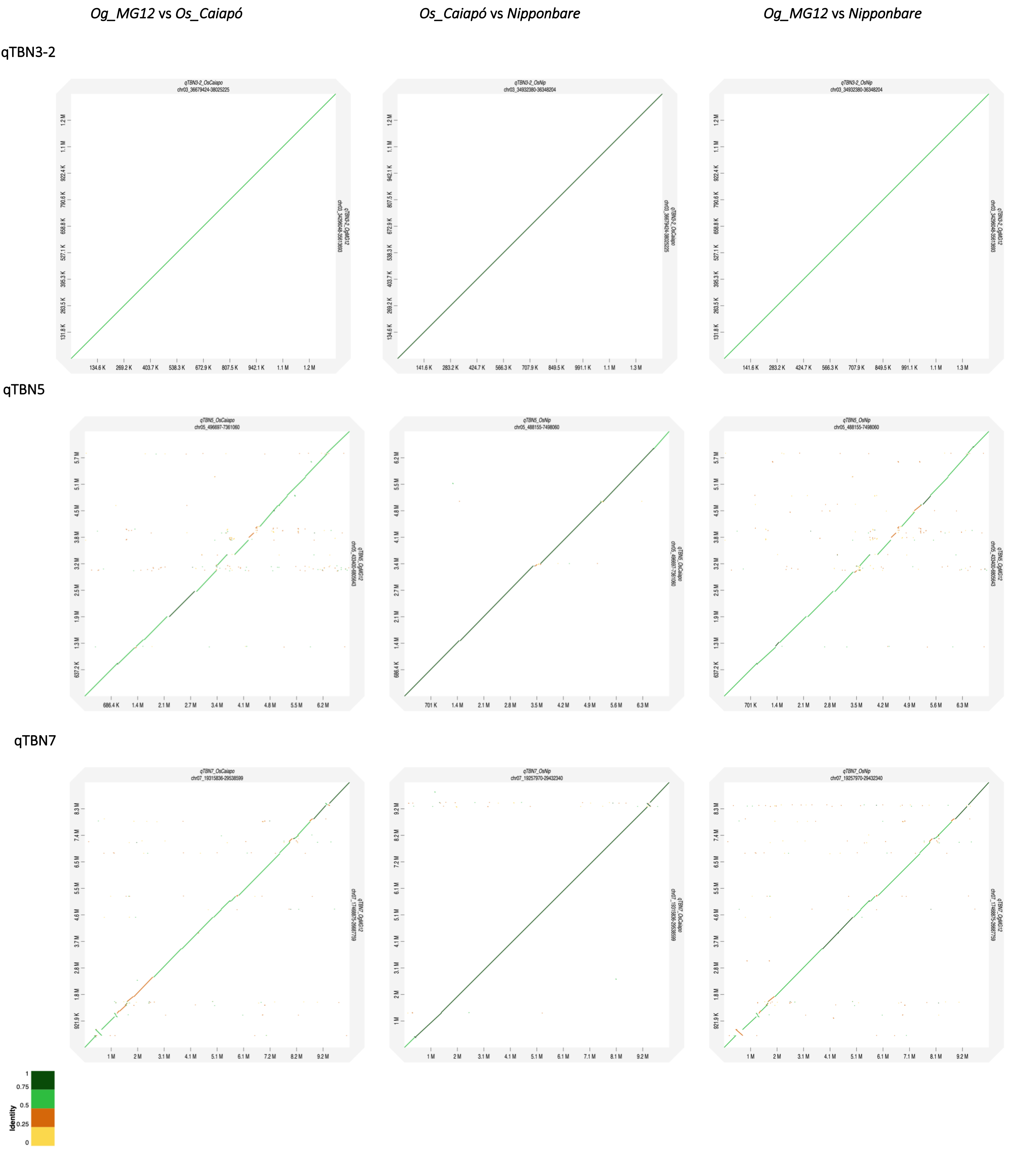

Additional file 2: Figure S5. Dotplots of the minimap2 alignment (implemented in D-GENIES web facilities) of panicle trait-related QTLs detected between the *Os_Caiapó* and *Og_MG12* genomes and between each of the latter aligned against *O. sativa* cv. *Nipponbare* as a reference genome. QTL coordinates in each genome are indicated on axis X and Y. Dot colors are relative to the identity value (I) which is a BLAST-like alignment identity (I=Number of bases, including gaps per number of matching bases in the bases). Correction to make in the figure (top right): Nipponabre -> Nipponbare

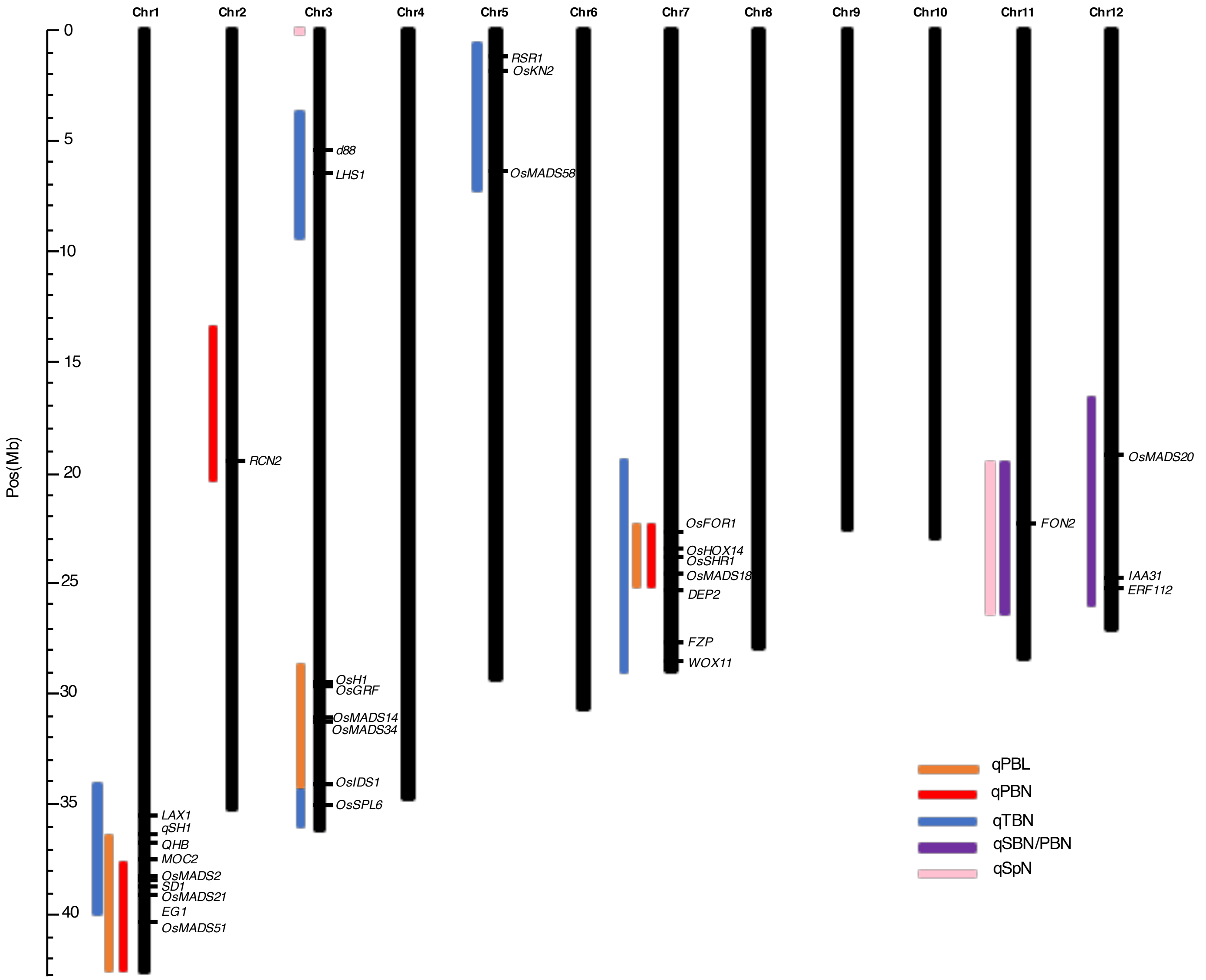

Additional file 2: Figure S6 : Physical map positions of detected QTLs and colocalizations with known genes related to branching and flowering.
